# Pre-existing partner-drug resistance to artemisinin combination therapies facilitates the emergence and spread of artemisinin resistance: a consensus modelling study

**DOI:** 10.1016/S2666-5247(22)00155-0

**Published:** 2022-09

**Authors:** Oliver J Watson, Bo Gao, Tran Dang Nguyen, Thu Nguyen-Anh Tran, Melissa A Penny, David L Smith, Lucy Okell, Ricardo Aguas, Maciej F Boni

**Affiliations:** a Medical Research Council Centre for Global Infectious Disease Analysis, Faculty of Medicine, Imperial College London, London, UK; b Centre for Tropical Medicine and Global Health, Nuffield Department of Medicine, University of Oxford, Oxford, UK; c Center for Infectious Disease Dynamics, Department of Biology, Pennsylvania State University, University Park, PA, USA; d Swiss Tropical Public Health Institute, Basel, Switzerland; e Department of Health Metrics Sciences, University of Washington, Seattle, WA, USA

## Abstract

**Background:**

Artemisinin-resistant genotypes of *Plasmodium falciparum* have now emerged a minimum of six times on three continents despite recommendations that all artemisinins be deployed as artemisinin combination therapies (ACTs). Widespread resistance to the non-artemisinin partner drugs in ACTs has the potential to limit the clinical and resistance benefits provided by combination therapy. We aimed to model and evaluate the long-term effects of high levels of partner-drug resistance on the early emergence of artemisinin-resistant genotypes.

**Methods:**

Using a consensus modelling approach, we used three individual-based mathematical models of *Plasmodium falciparum* transmission to evaluate the effects of pre-existing partner-drug resistance and ACT deployment on the evolution of artemisinin resistance. Each model simulates 100 000 individuals in a particular transmission setting (malaria prevalence of 1%, 5%, 10%, or 20%) with a daily time step that updates individuals' infection status, treatment status, immunity, genotype-specific parasite densities, and clinical state. We modelled varying access to antimalarial drugs if febrile (coverage of 20%, 40%, or 60%) with one primary ACT used as first-line therapy: dihydroartemisinin–piperaquine (DHA-PPQ), artesunate–amodiaquine (ASAQ), or artemether–lumefantrine (AL). The primary outcome was time until 0·25 580Y allele frequency for artemisinin resistance (the establishment time).

**Findings:**

Higher frequencies of pre-existing partner-drug resistant genotypes lead to earlier establishment of artemisinin resistance. Across all models, a 10-fold increase in the frequency of partner-drug resistance genotypes on average corresponded to loss of artemisinin efficacy 2–12 years earlier. Most reductions in time to artemisinin resistance establishment were observed after an increase in frequency of the partner-drug resistance genotype from 0·0 to 0·10.

**Interpretation:**

Partner-drug resistance in ACTs facilitates the early emergence of artemisinin resistance and is a major public health concern. Higher-grade partner-drug resistance has the largest effect, with piperaquine resistance accelerating the early emergence of artemisinin-resistant alleles the most. Continued investment in molecular surveillance of partner-drug resistant genotypes to guide choice of first-line ACT is paramount.

**Funding:**

Schmidt Science Fellowship in partnership with the Rhodes Trust; Bill & Melinda Gates Foundation; Wellcome Trust.

## Introduction

Worldwide adoption of artemisinin combination therapies (ACTs) against uncomplicated *Plasmodium falciparum* malaria began after WHO recommended ACTs as the first-line therapy in all malaria-endemic countries in 2005.^1^ Before 2005, artemisinin was primarily used in southeast Asia, both in monotherapies and in combination therapies. The emergence of artemisinin resistance in the Greater Mekong subregion has been largely attributed to this long history and increased use of artemisinin derivatives compared with Africa, particularly as a monotherapy. For example, despite the national switch to artesunate–mefloquine in 2000, 78% of all the artemisinin delivered in Cambodia in 2002 was used as a monotherapy.^2^ This uncontrolled use of artemisinin monotherapy increased the risk of artemisinin resistance emerging. Despite efforts to remove monotherapies from private markets and clinical use,^3^ the first documented cases of artemisinin resistance were observed in western Cambodia in 2008,^4^ although the resistant genotype had emerged years earlier and was already at high frequency in 2002.^5^ Currently, the major burden of artemisinin-resistant genotypes is confined to southeast Asia; however, independent emergence of artemisinin resistance has now been identified in Guyana^6^ and the island of New Guinea.^7^ The first observations of de-novo emergence of artemisinin resistance mediated through *P falciparum* isolates carrying mutations in the *kelch13* gene in Africa were made in Rwanda in 2020^8^ and in Uganda in 2021.^9^

The vast majority of artemisinin is currently administered in the form of ACTs. A key role of the non-artemisinin partner drug is to reduce parasite densities of emergent artemisinin-resistant genotypes. However, in epidemiological scenarios in which *P falciparum* is resistant to these partner drugs, ACTs might already be acting as de-facto monotherapies.^10^ Mutations conferring high-level resistance to the partner drugs piperaquine and mefloquine have spread in parts of the Greater Mekong subregion. In Africa, the most commonly used partner drugs are lumefantrine and amodiaquine, for which partial resistance has been observed in multiple settings.^11^ High levels of ACT use in areas with high frequencies of partner-drug resistance might, firstly, pose an increased risk of de-novo emergence and spread of artemisinin resistance and, secondly, create conditions in which imported artemisinin-resistant genotypes are rapidly selected for. By contrast, choosing ACT policy according to local partner-drug efficacy might reduce the spread of resistance.

**Research in context**

**Evidence before this study**

We searched the scientific literature using PubMed with the search terms “projection OR mathematical model” AND “malaria OR falciparum OR plasmodium” AND “resistance”, for English-language articles published between Jan 1, 2000 and June 30, 2022. We did not identify any modelling studies that used a consensus approach with multiple models to evaluate drug-resistance outcomes. Additionally, although the spread of antimalarial resistance has been simulated in a few individual-based mathematical modelling studies, only one other study used treatment-by-genotype efficacy estimates parameterised by matching to clinical trial data. This effort has only recently been possible with the increased availability of clinical trials and maps of resistance markers.

**Added value of this study**

In this study, a consortium of modelling groups estimated the speed of selection of artemisinin resistance under a range of epidemiological settings with different first-line artemisinin combination therapies and pre-existing frequencies of partner-drug resistance. Using a consensus approach with three established transmission models, our analysis showed that the early emergence and establishment of artemisinin resistance is facilitated by the presence of partner-drug resistance. This finding was robustly observed across all models and settings despite multiple differences between the three models used. In addition, a broad consensus was achieved in showing that most of this effect takes place in the early stages of partner-drug resistance evolution, as partner-drug resistant genotypes advance from 0 to 0·1 genotype frequency.

**Implications of all the available evidence**

The best available evidence indicates that early surveillance for partner-drug resistance is necessary to prevent the spread of artemisinin resistance, with low frequencies of partner-drug resistance facilitating the early emergence of artemisinin resistance. Higher-grade partner-drug resistance, as is observed for piperaquine-resistant genotypes, results in stronger selection of artemisinin resistance; consequently, dihydroartemisinin–piperaquine is the artemisinin combination therapy that is most susceptible to the process of partner-drug resistance accelerating the evolution of artemisinin resistance.

Historically, artemisinin-resistant genotypes in Cambodia arose in the 1990s in the absence of partner-drug resistance. Mefloquine resistance probably arose before artemisinin resistance on the border between Thailand and Myanmar in the late 1980s.^12^ In Uganda and Rwanda, artemisinin-resistant genotypes emerged in the presence of partial lumefantrine resistance with some known molecular markers for lumefantrine resistance at intermediate-to-high levels.^11^ In general, it appears that artemisinin resistance can emerge on backgrounds of partner-drug resistance or partner-drug sensitivity, a process that is dependent on the random nature of mutation and the specific drugs used in populations over long periods. In this Article, we evaluate the long-term effects of high levels of partner-drug resistance on the early emergence of artemisinin-resistant genotypes. We use a consensus approach taken in previous mathematical modelling studies^13^ and present results from three independently built, individual-based models of *P falciparum* malaria.

## Methods

### Model descriptions

Three individual-based stochastic models of malaria transmission and evolution were used to evaluate the evolution of artemisinin resistance under different pre-existing levels of ACT partner-drug resistance. The three models were developed by the Medical Research Council Centre for Global Infectious Disease Analysis at Imperial College London (London, UK),^14^ the Center for Infectious Disease Dynamics at Pennsylvania State University (PSU; University Park, PA, USA),^10^ and the Mahidol-Oxford Research Unit (MORU) affiliated with the University of Oxford (Oxford, UK).^15^ Each model simulates 100 000 individuals with a daily time step that updates individuals' infection status, treatment status, immunity, genotype-specific parasite densities, and clinical state.

Each model tracks the dynamics of clonal blood-stage *P falciparum* populations within individuals. A key common feature is that individuals can be infected with multiple clones simultaneously. Each parasite clone was described by a genotype that characterises its antimalarial resistance phenotype. These genotypes include the K76T locus in *pfcrt* (*P falciparum* chloroquine resistance transporter gene), N86Y and Y184F in *pfmdr1* (*P falciparum* multidrug resistance gene 1), C580Y in *pfkelch13*, copy number variation (CNV) of the *pfmdr1* gene, and CNV of the *P falciparum* plasmepsin 2 and 3 (*pfpm2–3*) genes. CNV was treated as a binary variable, distinguishing between single copies and multiple copies, resulting in 64 possible genotypes that were tracked. The models allowed for treatment of febrile malaria with three different ACTs: artemether–lumefantrine (AL), artesunate–amodiaquine (ASAQ), and dihydroartemisinin–piperaquine (DHA-PPQ). The same parameterisation,^16^ of 192 (3 × 64) treatment efficacies of three therapies on 64 genotypes, was used in all three models. Pleiotropy at the *pfcrt* and *pfmdr1* loci^11^ was accounted for with the effects of the *pfcrt* and *pfmdr1* genotypes on efficacy of both lumefantrine and amodiaquine. The C580Y locus was used as a proxy for artemisinin resistance, recognising that multiple *pfkelch13* mutations have been associated with a drop in artemisinin efficacy. The CNVs of the *pfpm2* and *pfpm3* loci were used as a proxy of piperaquine resistance, recognising the necessity of some *pfcrt* background mutations to achieve the maximally observed effect of piperaquine resistance (appendix pp 2–24).^17^

### Alignment

We conducted an alignment exercise to ensure model outputs were comparable across epidemiological scenarios. We aligned the three models' de-novo mutation rates so that the models reached 0·01 580Y allele frequency (ie, early emergence of artemisinin resistance; appendix p 29) after 7 years exactly, under a specified set of conditions: 100 000 individuals in a transmission setting with 10% all-ages malaria prevalence and 40% coverage with DHA-PPQ as first-line therapy (appendix p 30). This alignment ensured that one model did not generate a larger number of drug-resistant mutants simply because its mutation rate was higher. We also showed that the 0·01 allele-frequency threshold is insensitive to selection pressure for the mutation rates explored (appendix p 31), validating it as an appropriate cross-model alignment target. In addition, by using a common drug-by-genotype efficacy table,^16^ we ensured that the post-emergence treatment failure patterns are similar across all models (appendix pp 2–6). Treatment failure was defined as PCR-corrected recrudescence after 28 days, as recommended by WHO for surveillance of antimalarial efficacy.^18^ Crucially, by conducting our alignment this way, we followed similar model consensus exercise methodology to those used previously,^13^ by not aligning models by their outcome measures and instead evaluating the comparative impact of epidemiological scenarios in each model.

### Scenario evaluations

Each epidemiological scenario consisted of 100 000 individuals in a particular transmission setting (malaria prevalence of 1%, 5%, 10%, or 20%) without seasonality. We modelled varying access to antimalarial drugs if febrile (coverage of 20%, 40%, or 60%) with one primary ACT used as first-line therapy (DHA-PPQ, ASAQ, or AL). We explored five frequencies of pre-existing partner-drug resistance (0·00, 0·01, 0·10, 0·25, or 0·50), with partner-drug resistance allowed to spread in response to selection. For piperaquine, pre-existing resistance was defined by the frequency of genotypes with multiple copies of the plasmepsin genes. For amodiaquine and lumefantrine, we defined pre-existing resistance by the most resistant genotype in our parameterisation—ie, *pfcrt* 76T and *pfmdr1* 86Y Y184 for amodiaquine; and *pfcrt* K76, *pfmdr1* N86 184F, and double-copy *pfmdr1* genotypes for lumefantrine. 100 simulations were performed for each scenario, for a total of 18 000 simulations from all three models.

### Outcomes

The primary outcome measures was time to reach a particular resistance milestone: time to 0·25 frequency of the 580Y allele (*T*_Y,0·25_; artemisinin resistance). We refer to *T*_Y,0·25_ as the establishment time or the useful therapeutic life of artemisinins. Allele frequency was computed in a weighted manner, factoring in monoclonal and multiclonal infections (appendix p 2). We also report selection coefficients for the 580Y allele, defined via a sigmoidal fixation curve as follows:^19^

$$S_{i-j}=\frac{\log(\frac{j}{1-j})-\log(\frac{i}{1-i})}{T_{Y,j}-T_{Y,i}}$$

where *j* and *i* represent the frequency of the 580Y allele at different timepoints. In this Article, we present *S*_0·01–0·10_ to describe the strength of selection over the period between 0·01 and reaching 0·10 frequency and *S*_0·10–0·25_ for the period between 0·10 and reaching 0·25 frequency. These two periods of evaluation were chosen to show the early period of evolution from low frequencies (0·01) to moderate frequencies (0·10), in which random extinction becomes less probable, and the subsequent period to establishment (0·25), after which 580Y alleles are likely to undergo rapid selection and fixation (appendix p 29).

Comparisons of key model assumptions were explored in a sensitivity analysis, with the following assumptions varied: (1) fitness costs associated with resistance; (2) genotype-specific drug efficacies; (3) duration of asymptomatic infections; and (4) probability of developing clinical symptoms after an infection. These assumptions were chosen because varying them allowed us to explore different components within the transmission models at which the selective advantage of drug resistance manifests (appendix pp 36–39 and 44–46).

### Role of the funding source

The funders of the study had no role in study design, data collection, data analysis, data interpretation, or writing of the report.

## Results

**Figure 1:**
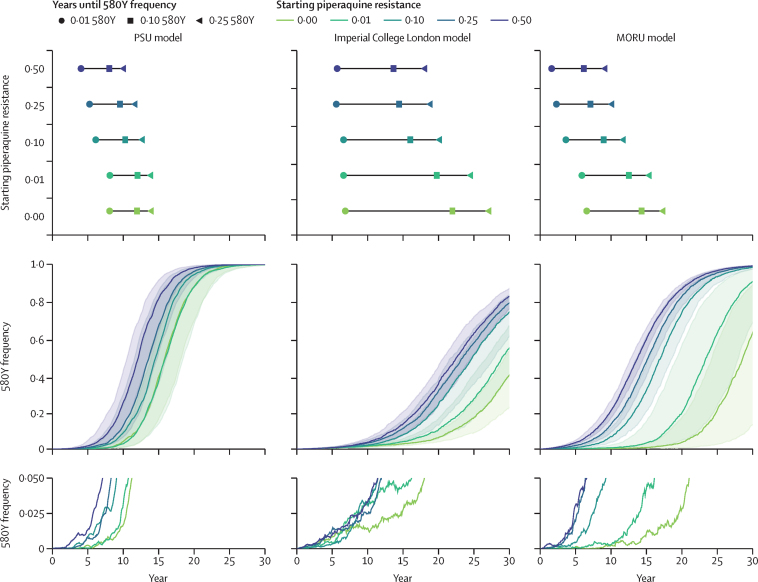
Artemisinin selection with respect to starting partner-drug resistance frequency In these scenarios, DHA-PPQ is used as the first-line therapy, with 40% population-level drug coverage and 5% malaria prevalence. Results are shown for five different starting piperaquine resistance frequencies (0·00 [green]–0·50 [purple]). The top row shows the median time to three resistance milestones for the five partner-drug resistance scenarios. The middle row shows the fixation pattern of artemisinin-resistant genotypes, characterised by the 580Y allele, with the median shown with solid lines and IQR shown with shaded bands. The bottom row shows the early patterns of emergence for five median simulations, where the median simulation is defined as the one whose time to 0·10 580Y allele frequency is the median time. DHA-PPQ=dihydroartemisinin–piperaquine. MORU=Mahidol-Oxford Research Unit. PSU=Pennsylvania State University.

Under long-term deployment of ACTs in malaria-endemic countries, artemisinin resistance emerges earlier if partner drugs are allowed to fail. For example, a scenario of 40% treatment coverage and 5% malaria prevalence resulted in artemisinin resistance emerging and fixing earlier under higher levels of pre-existing partner-drug resistance (figure 1). In these modelled scenarios, the recommended first-line ACT was DHA-PPQ, and only two mechanisms of genetic resistance were tracked—the *pfkelch13* 580 locus and copy number of the *pfpm2–3* genes. The average rate of selection was approximately equal under different frequencies of pre-existing piperaquine resistant genotypes because the relative fitness advantage of 580Y over C580 stays approximately constant as C580 alleles are replaced (appendix p 32). However, with pre-existing piperaquine resistance at higher levels, the early stochastic stages of artemisinin resistance emergence were less susceptible to random extinction (figure 1), enabling 580Y alleles to establish so-called escape velocity (sufficiently high frequency that the probability of extinction is low) earlier and replace drug-sensitive alleles more quickly. This difference between early-phase selection and late-phase selection can be observed by contrasting the approximately flat selection coefficients, with respect to pre-existing piperaquine resistance, when calculated during the *S*_0·10–0·25_ phase of selection against the sloped selection coefficients when calculated during the *S*_0·01–0·10_ phase of selection (appendix p 32).

**Figure 2:**
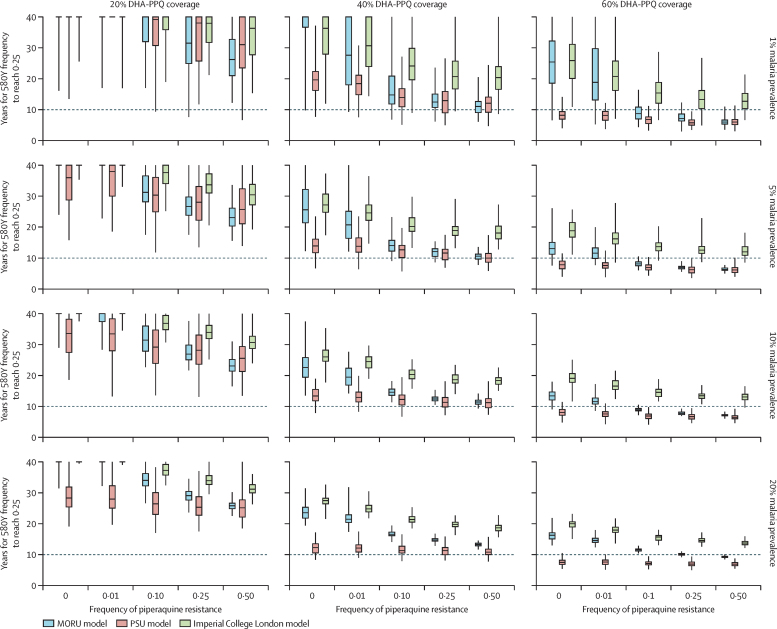
Number of years until 580Y allele frequency reaches 0·25 in regions with DHA-PPQ deployed as first-line therapy, starting from 0·00 580Y frequency Results for different coverage levels (three columns) and different prevalence levels (four rows) are shown. As the initial genotype frequency of piperaquine resistance increases, the time to 580Y establishment gets shorter. The box (median and IQR) and whisker (95% quantile range from 100 simulations) plots presented are censored box plots, with simulations only being run for 40 years. Values greater than 40 years contribute to the median, IQRs, and 95% ranges if calculable. DHA-PPQ=dihydroartemisinin–piperaquine. MORU=Mahidol-Oxford Research Unit. PSU=Pennsylvania State University.

Across transmission and coverage settings, all three models predicted quicker progression to high frequencies of artemisinin resistance when piperaquine resistance was high than when it was low. Under 40% ACT coverage with no pre-existing piperaquine resistance, the median time to establishment (580Y frequency of 0·25) across all four malaria prevalence settings was 26·0–36·3 years after introducing DHA-PPQ as a first-line treatment (Imperial College London model), 12·3–19·6 years after introduction (PSU model), and 22·6–40·0+ years after introduction (MORU model; figure 2). This duration includes the 7 years taken for 580Y alleles to reach 0·01 frequency (appendix p 30). The variation in durations is a feature of each model's construction, with each using different implementations of the epidemiological, entomological, and clinical aspects of malaria (appendix pp 3–6); the PSU model had a notably faster selection rate than the other models due to a different assumption in the way that the drug's half-life affects the probability of mutation (appendix p 27). When piperaquine resistance was present at an allele frequency of 0·10, 580Y establishment occurred 21·9–33·6% earlier than under no pre-existing piperaquine resistance in the Imperial College London model, 8·1–29·1% earlier in the PSU model, and 29·4–63·0% earlier in the MORU model (figure 2). The consistent reduction in establishment time across all models and four prevalence settings indicates that this reduction is a robust feature of *P falciparum* evolutionary dynamics in modern treatment contexts—namely, that early emergence and establishment of artemisinin resistance is facilitated by the presence of piperaquine resistance in settings where DHA-PPQ is used as first-line therapy.

**Figure 3:**
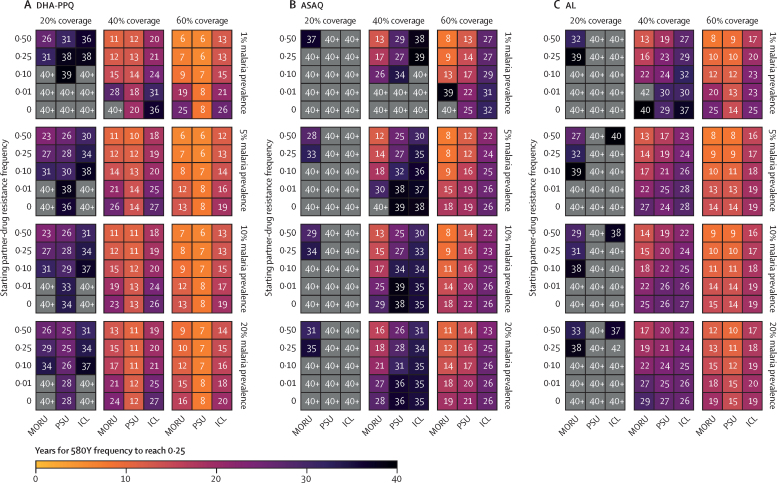
Median time (*T*_Y,0·25_) for artemisinin-resistant genotypes to reach a frequency of 0·25, under different pre-existing allele frequencies for partner-drug resistance Simulations were evaluated over a 40-year period with median times taking longer than 40 years indicated in grey (40+ years). Times are shown for each model at four different malaria prevalences (1%, 5%, 10%, and 20%) and three different treatment coverages (20%, 40%, and 60%). In all settings and across all models, a decrease in time to 0·25 artemisinin resistance frequency was observed with increasing initial partner-drug resistance. AL=artemether–lumefantrine. ASAQ=artesunate–amodiaquine. DHA-PPQ=dihydroartemisinin–piperaquine. ICL=Imperial College London. MORU=Mahidol-Oxford Research Unit. PSU=Pennsylvania State University.

**Figure 4:**
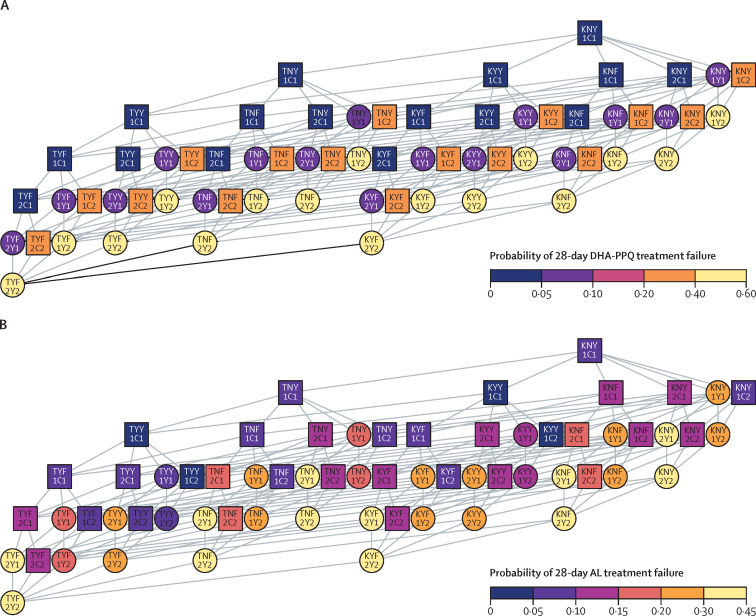
Comparison of fitness landscapes of DHA-PPQ and AL Network diagrams connect genotypes that are one mutation apart and arrange all genotypes from the wild type (top) to multi-allelic resistant types (bottom); each horizontal level corresponds to one additional genetic mechanism (mutation or copy number variation) of resistance. The genotype is labelled for each node (eg, the wild type at the top, KNY1C1, contains *pfcrt* K76, *pfmdr1* N86 and Y184, one copy of *pfmdr1, pfkelch* C580, and one copy of the *pfpm2* and *pfpm3* genes). The 28-day probability of treatment failure for each parasite genotype is indicated by the discrete colour of the nodes in the network for DHA-PPQ (A) and AL (B), which is detailed in the colour bar. Yellow shades show increased resistance (increased probability of treatment failure). Genotypes with the 580Y artemisinin-resistant allele are shown with circles, and genotypes with the wild-type C580 allele are shown with squares. The network highlights the comparatively more complex fitness landscape associated with AL resistance (16 different treatment failure phenotypes) than with DHA-PPQ resistance (4 different phenotypes). AL=artemether–lumefantrine. DHA-PPQ=dihydroartemisinin–piperaquine.

The rate of evolution of artemisinin resistance differed among the three modelled ACTs (figure 3). The difference in selection patterns can be explained by the differential efficacy of each ACT as resistance mutations accumulate to the partner drugs, as well as by the different number of genetic mechanisms required to achieve maximum levels of resistance (figure 4; appendix p 41). On average, DHA-PPQ treatment efficacy (28-day parasite clearance) drops the most, from the wild-type genotype (97%) to the maximally resistant genotype (42%; appendix p 24), followed by AL (97% to 57%) and then ASAQ (98% to 74%). Hence, for all three models, resistance evolution under DHA-PPQ yielded the greatest selection coefficients (appendix p 33). With no pre-existing partner-drug resistance, a malaria prevalence of 5%, and 40% treatment coverage, median establishment time across all models was 23·8 years (IQR 16·2–28·3) for DHA-PPQ, 37·1 years (31·5–44·5) for ASAQ, and 27·3 years (23·8–32·5) for AL (table; figure 3). Time until establishment of artemisinin resistance varied according to first-line therapy, malaria prevalence, and treatment coverage (figure 2). Evolution was always faster under higher treatment coverage, in all models and scenarios (figure 3). In our modelled scenario runs, resistance evolution was generally but not always faster at higher prevalence levels (figure 3, appendix p 35). This general trend of resistance evolution occurring more quickly at higher prevalence levels results from more de-novo mutations and shorter parasite generation times than at lower prevalence levels. This observation was most pronounced in scenarios explored at a malaria prevalence of 1%, which exhibited higher variance in emergence times leading to (on average) longer establishment times for 580Y than at higher prevalences. Additionally, the mutational route (partner-drug or artemisinin resistance emerging first) towards 580Y establishment was also different between ACTs (appendix p 34). DHA-PPQ was predicted to select for partner-drug resistance first, whereas AL selected for 580Y before partner-drug resistance. For ASAQ, both mutational routes were probable.

**Table:** Years to 0·25 580Y frequency under 40% treatment coverage, 5% malaria prevalence, and different starting frequencies of partner-drug resistance

	**Years till 0·25 580Y frequency (PD_0_)**	**Reduction in establishment time (or artemisinin UTL) when comparing with PD_0_ scenario**				**Years lost per log_10_ increase in starting partner-drug frequency**
		PD_0·01_	PD_0·10_	PD_0·25_	PD_0·50_	
**MORU model**						
DHA-PPQ	25·6 (21·4 to 32·2)	20·6% (13·7 to 27·0)	47·3% (41·6 to 53·0)	55·3% (49·7 to 60·7)	60·6% (54·8 to 66·2)	6·4
ASAQ	41·7 (30·5 to 53·3)	21·8% (12·3 to 32·5)	54·6% (46·9 to 62·4)	63·9% (56·2 to 71·5)	69·9% (62·3 to 77·3)	12·1
AL	27·2 (22·4 to 34·4)	19·1% (12·0 to 26·3)	38·4% (32·4 to 44·6)	48·4% (42·2 to 53·7)	54·9% (49·3 to 60·7)	5·9
**PSU model**						
DHA-PPQ	13·9 (11·7 to 16·1)	−1·3% (−8·4 to 5·5)	12·6% (5·9 to 19·0)	18·3% (12·5 to 24·4)	26·6% (21·0 to 32·4)	2·2
ASAQ	38·8 (31·0 to 44·0)	−0·5% (−7·6 to 6·4)	15·7% (9·9 to 22·1)	26·7% (20·6 to 33·2)	30·3% (24·8 to 36·4)	7·0
AL	24·5 (21·4 to 30·8)	0·0% (−6·9 to 6·7)	13·1% (5·9 to 19·6)	23·5% (17·7 to 29·3)	32·3% (26·8 to 37·8)	4·8
**ICL model**						
DHA-PPQ	27·1 (24·8 to 30·7)	9·3% (5·3 to 13·2)	24·3% (20·7 to 27·9)	30·6% (27·1 to 34·0)	33·0% (29·3 to 36·7)	3·9
ASAQ	38·3 (33·2 to 42·0)	2·1% (−1·5 to 5·9)	3·3% (−0·5 to 7·2)	10·0% (5·9 to 13·7)	18·4% (14·3 to 22·2)	3·2
AL	28·0 (24·1 to 31·7)	1·1% (−3·7 to 6·1)	5·5% (0·4 to 10·3)	13·0% (8·0 to 17·5)	16·5% (12·2 to 21·0)	2·5

Median years (IQR) are presented for scenarios with no starting partner-drug resistance (PD_0_) before showing the mean percentage difference in years to 0·25 580Y frequency for each frequency of starting partner-drug resistance explored. Censored times above 40 years were inferred using a Weibull distribution to describe time-to-event values (results for other prevalence levels are shown in the appendix [pp 49–51]). AL=artemether–lumefantrine. ASAQ=artesunate–amodiaquine. DHA-PPQ=dihydroartemisinin–piperaquine. ICL=Imperial College London. MORU=Mahidol-Oxford Research Unit. PSU=Pennsylvania State University. UTL=useful therapeutic life.

Establishment times for 580Y showed a clear pattern of occurring earlier under higher partner-drug resistance (table). A magnitude increase in the starting partner-drug resistance frequency (eg, 0·01 to 0·10) was associated with a range of 2–12 years of lost artemisinin efficacy. Ranges were taken across all prevalence levels, first-line therapies, and models. The number of years of lost artemisinin efficacy was approximately log-linear with respect to increases in partner-drug resistance (appendix p 35). Consequently, a major part of the reduction in establishment time was observed after the first 0·10 increment of genotype frequency of partner-drug resistance.

As several model assumptions are known to affect evolutionary dynamics but are difficult to validate or estimate with field data, we conducted sensitivity analyses of the resistant genotypes' fitness costs, the genotype-specific drug efficacies, the duration of infection of asymptomatic carriage, and the probability of progressing to symptoms after an infectious bite (appendix pp 36–39). Although evolutionary rates were affected by these changes—eg, higher fitness costs and a lower probability of symptoms led to slower drug-resistance evolution—the association between higher pre-existing allele frequencies of partner-drug resistance and earlier 580Y establishment was shown to be robust under all scenarios examined. This finding was similarly robust when we introduced uncertainty into the relative associations assumed between parasite genotype and drug efficacy (appendix pp 44–45).

## Discussion

Drug-resistance surveillance efforts have recently been focused on understanding the emergence, spatial spread, and evolution of artemisinin-resistant genotypes. After the discovery of molecular markers underpinning artemisinin resistance in southeast Asia,^5^ several studies were done to investigate the prevalence of these markers in Africa. By contrast, resistance to partner drugs has received comparatively less attention, despite ACTs being used in epidemiological settings with endemic resistance to amodiaquine, lumefantrine, piperaquine, and mefloquine. Our results show that focusing surveillance on partner-drug resistance is needed to slow the spread of artemisinin resistance.

In this Article, we show that the emergence and spread of partner-drug resistance gradually erodes both ends of an ACT's useful therapeutic life, via immediate reductions in efficacy due to lower partner-drug pharmacodynamic activity and by shortening the period that ACTs can be used at full efficacy before artemisinin-resistant genotypes are established. These findings are consistent with previous pharmacokinetic and pharmacodynamic analyses of drug action.^20^ Using three independently calibrated, stochastic, individual-based malaria models, the primary evolutionary effect appeared to occur at low 580Y allele frequencies. During this early emergence period, artemisinin-resistant genotypes are able to establish more quickly when appearing on a genetic background of partner-drug resistance. An analysis of selection coefficients during the establishment phase (580Y frequencies >0·10) also showed an additional but smaller effect of stronger selection of 580Y alleles when appearing alongside partner-drug resistance mutations (appendix p 32).

Substantial reductions in the establishment time for artemisinin resistance were observed across all models after an increase in partner-drug resistance frequency from 0·00 to 0·10. These effects are of most practical consequence for DHA-PPQ, as evolution of artemisinin-resistance was fastest (in all three models) in scenarios in which DHA-PPQ was deployed as first-line therapy. CNV at the plasmepsin loci corresponded to larger drops in ACT efficacy^21^ than those observed for lumefantrine-resistant and amodiaquine-resistant genotypes.^16^ However, this finding was dependent on the assumed treatment efficacies for each genotype (appendix p 24), which were parameterised from a model-based approach leveraging genotype and therapeutic efficacy data.^16^ Consequently, we explored the robustness of our findings by randomly varying the treatment efficacies of each drug independently (appendix p 44), while maintaining qualitative associations between genotypes and treatment efficacies known from the literature—eg, AL treatment efficacy was lower for strains with *pfmdr1* N86 than for strains with 86Y.^22^ Evolution of artemisinin resistance was still fastest with DHA-PPQ treatment; however, the variance in establishment times increased such that, in the most extreme variations of treatment efficacy, 580Y was selected for more quickly by AL than by DHA-PPQ (appendix p 45). A second reason for the earlier emergence of genotypes fully resistant to DHA-PPQ is that, in all three models, they required only one mutation and one CNV, whereas complete resistance to ASAQ and AL required three mutations, with smaller fitness differences associated with each individual mutational step. To confirm these effects, we did further sensitivity analyses in which the minimum and maximum treatment efficacy were similar for all three ACTs but the genetic associations and relative treatment efficacy against different genotypes were maintained (appendix p 46). In these scenarios, DHA-PPQ continued to select fastest for the maximally resistant genotype (appendix p 47).

Additional evolutionary mechanisms will play a role in navigating the multidrug resistance pathway to ACT resistance (figure 4). In our analyses, we predicted that DHA-PPQ would select for partner-drug resistance first, whereas AL would select for artemisinin resistance before partner-drug resistance; for ASAQ, either mutational route was probable (appendix p 34). However, it is important to note that there are multiple mechanisms with largely unknown parameters that might impact these predictions. Firstly, clonal competition probably slows down the emergence of drug resistance, with new genotypes having to compete within the host in multiclonal infections (appendix p 39). This competition is important if resistance incurs a fitness cost, meaning newly emerging drug-resistant parasites might be outcompeted by fitter drug-sensitive clones when drug levels are below inhibitory concentrations. Secondly, the likelihood of mutation events occurring in the absence of drug pressure and being onwardly transmitted will affect the generation of additional partner-drug resistant genotypes and impact the speed of selection due to increased interclonal competition in the population (appendix p 41). Thirdly, genetic recombination can act to both unite and break apart multi-genic resistant genotypes. Population genetics approaches are divided on whether recombination speeds up or slows down the arrival of multidrug resistant genotypes.^23^, ^24^ Our modelled patterns of resistance evolution did not show a consistent association with transmission intensity, suggesting that monitoring efforts should be supported wherever possible. Although low-transmission regions have historically been associated with drug-resistance emergence, our findings are consistent with both empirical and theoretical work suggesting a non-monotonic association between malaria prevalence and resistance evolution.^25^, ^26^ Finally, seasonal malaria transmission, rapidly changing prevalence, and bottleneck effects do affect the trajectories of genotypes under selection.^27^, ^28^ A more precise assessment of the risk of partner-drug resistance would require the use of country-specific epidemiological scenarios.

Our structured model comparisons have several limitations. Firstly, drug-resistant genotypes (across pathogens) generally carry fitness costs and our assumed fitness costs might not have been accurate. In-vivo fitness costs are hard to estimate, often only becoming known after the withdrawal of a first-line therapy or by relying on in-vitro genetic studies or feeding-assay approaches to characterise the relative fitness and transmissibility of resistant clones.^29^ Second, the incremental fitness benefits of drug-resistance mutations are equally difficult to parameterise as therapeutic efficacy studies are not powered to measure efficacy on specific genotypes. A single parameterisation of genotype-specific drug efficacies was used in our analysis (even though there is likely to be considerable variation in these estimates), which was based on one-compartment pharmacokinetic assumptions. This parameterisation simplified the mode of action of the partner drugs, which have more complex pharmacokinetic properties. However, a sensitivity analysis on these efficacy values did not alter our general conclusions on partner-drug resistance facilitating artemisinin-resistance emergence (appendix p 37), but did remind us that there is substantial uncertainty in the absolute measures presented in this study and some uncertainty in the order of emergence events when multiple alleles confer resistance to the same drug (appendix p 34). Despite this uncertainty, we were encouraged to find that our observed selection coefficients for each ACT fell within the range of selection coefficients estimated in a recent review (appendix p 33).^19^ Third, model consensus studies are fairly new and choosing parameters to harmonise across models that are not proximally and immediately affecting the outcome measures is challenging. In this study, we chose to align the de-novo mutation rate because the de-novo *P falciparum* mutation rate and within-host competition of new mutants are unknown. Consequently, the mutation rates chosen and the time to 0·01 allele frequency might not be representative; however, this alignment allowed for meaningful model comparisons for the time until 0·25 allele frequency.

As a means of basic public health investment, a renewed focus should be placed on early molecular surveillance for partner-drug resistant genotypes. As in all expanded surveillance scenarios, the health-economic equation balancing monetary costs against successful delays of the onset of resistance is the crucial one to assess. This general cost–benefit question of investing in early drug-resistance surveillance at low genotype frequencies requires more attention in the malaria literature. Although public health concern typically manifests only after partner-drug resistance is common, the work we present in this Article suggests that early detection of and pre-emptive action against partner-drug resistance^30^ would have the benefit of delaying partner-drug resistance, artemisinin resistance, and treatment failure, all at once.

## Declaration of interests

We declare no competing interests.
