# Supplementary material for: Pre-existing partner-drug resistance to artemisinin combination therapies facilitates the emergence and spread of artemisinin resistance: a consensus modelling study
Source: Lancet Microbe. 2022 Sep;3(9):e701–10. doi: 10.1016/S2666-5247(22)00155-0 (PMC9436785; doi:10.1016/S2666-5247(22)00155-0)
Supplement: Supplementary appendix [file mmc1.pdf]

THE LANCET
Microbe

Supplementary appendix

This appendix formed part of the original submission and has been peer reviewed. We post it as supplied by the authors.

Supplement to: Watson O J, Gao B, Nguyen T D, et al. Pre-existing partner-drug resistance to artemisinin combination therapies facilitates the emergence and spread of artemisinin resistance: a consensus modelling study. *Lancet Microbe* 2022; published online Aug 2. https://doi.org/10.1016/S2666-5247(22)00155-0.

Pre-existing partner-drug resistance facilitates the emergence and spread of artemisinin resistance: a modelling study

Supplementary Material

Supplementary Material **1**
Supplementary Methods 2
1 Model Descriptions 2
2 Model Specific Extensions 7
Imperial Model 7
MORU Model 15
PSU Model 20
3 Treatment efficacy on specific genotypes 23
4 Sensitivity Analyses and Outcome Measures 25
5 Impact of altering relationship between genotypes and drug efficacy 27
5.1 Uncertainty in estimates of drug efficacy by genotype 27
5.2 Comparable drug efficacy by genotype across ACT 27
Supplementary Figures 29
Supplementary Tables 48
Supplementary References 52

Supplementary Methods

1 Model Descriptions

Three individual-based microsimulations were used to compare patterns of artemisinin-resistance evolution in different contexts of prevalence, treatment coverage, and pre-existing partner-drug resistance. The three models are called (1) the ‘Imperial Model’, developed at the MRC Centre for Global Infectious Disease Analysis, Faculty of Medicine, Imperial College London; (2) the ‘MORU Model’, developed at the Mahidol-Oxford Research Unit (MORU), Nuffield Department of Medicine, University of Oxford; and (3) the ‘PSU Model’, developed at the Center for Infectious Disease Dynamics (CIDD), Department of Biology, Pennsylvania State University. The models all run as daily time-step discrete-event simulations of individuals (humans) who can be infected with *Plasmodium falciparum* malaria and subsequently pass on their infection to other individuals in the simulation. The outputs of model runs and downstream analysis code is publicly available as a reproducible research compendium at https://github.com/OJWatson/art_resistance_consensus_modelling.

An overview of the comparisons between each model are detailed in Table S1. Further methodological details of the individual models and extensions made to previously published models are described below for each model.

Supplementary Table 1. Comparative model features

	Imperial Model	PSU Model	MORU Model
**Key representative publications**	Original Imperial model (Griffin et al. 2016, 2010). Extensions made for modelling parasite genetics in MBE (Watson et al. 2021).	Multiple first line therapies study in Lancet Global Health (Nguyen et al. 2015) and therapeutic efficacy by genotype study bioRxiv preprint (Nguyen et al. 2021))	Mass drug administration study in eLife (Gao et al. 2020)
**Accessibility (Either Github or description of code availability etc.)**	Model code is open source and freely available at www.github.com/OJWatson/magenta . In this study, magenta version 1.3.0 was used (Watson, Verity, and FitzJohn 2021)	Code is open source at https://github.com/bonilab/malariaibm-MMC-WP2-partnerdrugresistance (version 3.2)	Code is open source at https://github.com/ATOME-MORU/malaria-model-v1.0-20.3.19.
**Seasonality**	Yes; not used in present analyses but implemented through changing mosquito abundance.	Yes; not used in present analyses.	Yes; not used in present analyses.
**Heterogeneity in exposure**	Yes	Yes	Yes
**Blood-stage parasite densities modelled**	Not explicitly. Parasite densities are approximated by tracking a parasite in terms of the infection status that the parasite causes in the absence of superinfection (symptomatic, asymptomatic, sub-patent). These states are then used to calculate the relative probability of different strains being passed on when a human infects a mosquito. See (Watson et al. 2021) for further detail.	Yes	No
**Parameterization for clinical incidence**	Fitted to cross-sectional age-incidence data from 23 sites in Africa capturing differences between active and passive case detection (Griffin, Ferguson, and Ghani 2014).	Calibrated to data sets assembled for five different African studies measuring age-specific clinical incidence and EIR. No formal model fitting done.	We parameterised the age dependent risk of infection during the first 10 years of life using data from 8 endemic countries in sub-Saharan Africa (Aguas et al., 2008). Given the age-dependent force of infection function, we fit the model-predicted age-dependent clinical disease incidence against two separate data sets from SE Asia (one with age, one without). Details in ((Gao et al. 2020).

**Parameterization for severe disease and mortality incidence**	Severe disease model (Griffin et al. 2015) fitted to data from northern Tanzania (Reyburn et al. 2005) and to severe disease vs. prevalence relationship from data of multiple sites (Marsh and Snow 1999). Mortality due to malaria is based on Africa-wide data from verbal autopsy and parasite prevalence (Rowe et al. 2006).	Mortality only. No tracking of severe malaria. Treatment failures and untreated cases are associated with 4% mortality (age groups 0-1), 2% mortality (age groups 2-5), 0.4% mortality (age groups 6-10), and 0.1% mortality (age groups 11 and older); see section 6 of supplement to (Nguyen et al. 2015). Mortality is zero for successfully treated malaria cases.	Of those clinical infections, we estimate the proportion that results in severe disease and hospitalisation (by age) from data in (Marsh and Snow 1999). The (Marsh and Snow 1999) dataset also informs the mortality rates per hospitalised case per age group. Maximum mortality rates in hospitalised children was 5% (<2 year olds). Older children had a greater treatment success rate with mortality rates of less than 0.5%.
**Drug interventions – PK- PD Drug action etc**	Slow clearance, treatment failure incorporated. PKPD not explicitly incorporated in the transmission model. PKPD are modelled separately and their outputs incorporated as parameters that determine the probability of slow parasite clearance or late parasitological failure as defined in (Slater et al. 2016).	Single compartment PK model used, with daily parasite killing, as a function of drug concentrations and parasite genotypes, for PD model.	Includes slow clearance, treatment failure, appearance and spread of resistance. Parasite clearance modelled using PD data from parasite clearance studies, with daily parasite killing, as a function of initial drug concentration, drug IC50 and parasite genotype.
**Genotype tracking and sensitivity to drugs**	Yes, four key resistance loci and two copy number variants tracked. 64 total genotypes.	Yes, four key resistance loci and two copy number variants tracked. 64 total genotypes.	Yes, four key resistance loci and two copy number variants tracked. 64 total genotypes.
**Vector control Interventions**	LLIN, IRS, Larval control (larviciding & pupaciding), and ivermectin.	Indirect vector control only, via transmission parameter that determines the daily amount of biting.	Yes – LLIN, IRS, Larval Control
**Treatment interventions**	Treatment of clinical disease and severe disease, by specified drug and diagnostic.<br>Mass screen and treat, IPTi, IPTc/SMC and IPTp/IST for separate pregnancy model	Yes, many types of drug policies such as multiple first-line therapies, cycling, adaptive cycling, triple therapy, mass drug administration, and private-market drug sales.	Yes treatment of clinical disease, MDA, MSAT, adjuctive primaquine, TME, iPT
**Treatment seeking and drug coverage**	Explored in the sensitivity analysis	Explored in the sensitivity analysis	Explored in the sensitivity analysis
**Spatial dynamic model**	Present analysis run in a single location.	Present analysis run in a single location.	Present analysis run in a single location.
**Super-infections, co-infections, multiplicity of infection**	Multi-strain model that tracks the multiplicity of infection arising from both superinfection and cotransmission events, in which a single mosquito transmits multiple sporozoites. Mosquitoes pass on an assumed geometric mean of 10 parasites during an infectious bite, with 21%	Yes, tracked explicitly as coinfections arising only from superinfection events.	Yes, each human individual can carry up to 10 different parasite populations (one per inoculum). Each inoculum is considered to be a clonal population upon emergence from the liver, with one parasite acquired from each infectious bite.

	surviving to produce a liver stage infection (21% parameterised in (Watson et al. 2021)).		
**Heterogeneity in Exposure**	Exposure varies both by age and between individuals.	Exposure varies both by age (v3.0.4) and between individuals.	Exposure varies both by age and between individuals.
**Duration of infection**	Duration of infection is "Erlang-like" distribution (convolution of exponential distributions), with overall duration calibrated to malariatherapy data.	Not drawn from a predetermined distribution. The duration of infection is determined by explicit modelling of parasitaemia and how drugs and the immune system act on parasites. Calibrated to malariatherapy data. Durations of infection in the model range from 60 to 281 days.	Asymptomatic infection duration based on malariatherapy data and clinical follow-up data from endemic areas, as well as drug efficacy data (PD). Also estimated from a set of 8 Endemic areas from Sub-Saharan Africa (Aguas et al 2008).
**Clinical disease and history of exposure**	A proportion of infected individuals go on to develop clinical disease (parameterised in (Griffin, Ferguson, and Ghani 2014)). Immunity to clinical disease develops with exposure and age, and also has a maternally acquired component.	A proportion of infected individuals go on to develop clinical disease. Immunity to clinical disease develops with exposure and age, and also has a maternally acquired component.	Proportion developing clinical disease depending on cumulative immunity from prior exposures and immunity level (indicator of recent exposure).
**Decay of natural immunity**	Exponential decay of naturally acquired immunity (parameterised in (Griffin, Ferguson, and Ghani 2014)).	Exponential decay of naturally acquired immunity.	Exponential decay of naturally acquired immunity as estimated in (Aguas et al. 2008)
**Infectiousness and gametocyte models**	Human infectiousness to mosquitos is dependent on the infection status of the human (clinical, asymptomatic, sub-patent) and if asymptomatic also determined by their immunity determining their probability of being detected by microscopy (parameterised in (Griffin, Ferguson, and Ghani 2014)).	Human infectiousness to mosquitos is a function of asexual parasite density, with a time lag built in to model the fact that infectious gametocytemia lags asexual parasitaemia..	Infectiousness depends on lagged development of sexual stage parasites and seasonal transmission equation from fitting to incidence data. It is informed by parasite density in an indirect way: clinical individuals are assumed to have a higher mean infectiousness compared to asymptomatic individuals as they carry higher parasite density loads.
**Entomological models**	Vector control interventions modelled through altering the life expectancy of a mosquito, altering the rate of anthrophagy. (described and parameterised in (Griffin et al. 2016)).	11-day lag built in so that FOI today depends on the biting done by mosquitoes 11 days ago. No other entomological features built in.	Full IBM component to mosquito dynamics. For this exercise we use a simplified version where only infectious mosquitoes are tracked individually. .
**Recombination model**	Multiple parasites taken up by mosquito to form *n* oocysts, which undergo recombination to yield up to 4xn genotypes of sporozoites in the mosquito. No interrupted feeding by	Recombination can occur in a mosquito bite on a multi-clonal host. Parasites are taken up by the mosquito in proportion to their parasite density. A full recombination table is built for all possible forces of infection resulting from this host's	No recombination. No interrupted feeding.

	single mosquito on multiple humans.	contribution to the next generation of infectious sporozoites, according to the normal rules of Mendelian genetics. No interrupted feeding allowed in present analyses.	
**Mutation model**	Back mutation present. Mutation can occur during treatment and in the absence of treatment. 1 rate for both calibrated during the calibration exercise.	No back mutation. Mutation can occur during treatment only when the mutation confers a resistance benefit to the current treatment.	No back mutation. Mutation can occur during treatment (higher rate), and in the absence of treatment (lower rate). The higher rate was calibrated during the calibration exercise.
**Stochasticity**	All model components are stochastic and described by defined probability distributions in (Watson et al. 2021). The only non stochastic elements are the following three delay terms (delay from mosquito oocyst formation to rupturing, delay from liver stage infection to clinical infection, and delay from clinical infection onset to gametocytogenesis), which are modelled as fixed durations.	All model components are stochastic and described by defined probability distributions defined in (Nguyen et al. 2015) and (Nguyen et al. 2021). The only non stochastic elements are the delay from mosquito oocyst formation to rupturing, which is modelled as a fixed duration.	All model components are stochastic and described by defined probability distributions defined in (Gao et al. 2020).
**PfPR range models calibrated against.**	Calibrated for prevalence ranges >= 1%, spanning EIR ranges from 0.5 - 200 (Griffin et al. 2010).	Immunity-symptom relationship calibrated to data sets where PfPR > 5%, across an EIR range of 10 to 200 (Nguyen et al. 2015).	Calibrated based on 8 data sets with $PfPR_{2\text{-}10}$ minimum of 2% (Aguas et al. 2008).

2 Model Specific Extensions

Imperial Model

The base model has been extensively described in (Watson et al. 2021). Briefly, it is an individual based, discrete time, stochastic model, with individual mosquitoes explicitly modelled, originally developed for characterising the impact of transmission intensity on neutral genetic diversity.

In overview, the transmission model considers people to exist in one of six infection states: susceptible (S), clinical disease (D), clinically diseased and receiving treatment (T), asymptomatic infection (A), protective state of prophylaxis (P), and subpatent infection (U). Individuals are born into the susceptible class and possess a level of maternal immunity that decays exponentially over the first 6 months. The model uses a fixed day time step, with individuals each day experiencing a force of infection that depends on their level of immunity, individual-level biting rate, and the number of infectious mosquitoes. If an individual is bitten by an infectious mosquito and infected, they then progress through a 12-day latent period either developing clinical disease or an asymptomatic infection dependent on their level of acquired immunity from previous infections. Individuals that develop clinical symptoms have a fixed probability of seeking treatment (the treatment coverage). If the treated individual is successfully treated, they then enter a protective state of prophylaxis, before returning to being susceptible, with the duration of prophylaxis dependent on the half life of the antimalarial. Individuals that did not receive treatment initially progress to an asymptomatic infection, followed by a subpatent infection before eventually clearing infection fully and returning to susceptible. All infected individuals that are not in the prophylactic state or the clinically diseased state are also susceptible to superinfection.

The adult stage of mosquito development is also modelled individually, with adult mosquitoes beginning life susceptible to infection. Mosquitoes seek a blood meal on the same day they are born and every 3 days after that until they die. Infected mosquitoes pass through a latent infection stage that lasts 10 days before becoming onwardly infectious to humans.

Parasites are modelled as discrete populations that result from an infection event associated with a mosquito or a human. There is no assumed maximum number of parasites, with individuals assumed to clear strains on the day that they would have moved from a subpatent infection to susceptible for the strain considered, i.e. each acquired strain follows an assumed trajectory in parasitaemia representative of a normal infection cycle. Acquired strains thus move "infection state" independently of the human's infection state. For example, a given individual is infected on day 0 and develops an asymptomatic infection. The individual is scheduled to become a subpatent infection on day 200, but they were bitten on day 150 and developed clinical symptoms and moved to state D. When this happens, the parasite density of the strain acquired on day 0 does not change and this strain will become a subpatent strain on day 200. After the parasite has moved to become a subpatent strain, the day at which the strain would have been cleared, i.e. the individual would have moved from state U to S if they had not been superinfected on day 150, is drawn and assigned to the parasite. This tracking of parasites allows for the relative parasitemias of each acquired strain to be calculated.

Each asexual parasite is represented by a genetic barcode, which is a series of 0s and 1s related to specific genetic markers. During the human to mosquito infection, multiple asexual parasites are sampled from the infected human, which result in the formation of multiple of oocysts that each yield up to 4 genetically different sporozoites resulting from recombination. During the mosquito to human infection, multiple sporozoites are onwardly transmitted. Full details of human, mosquito and parasite model components are detailed in the supplement of (Watson et al. 2021), which was used to model neutral genetic patterns

Below, we detail the extensions made for simulating antimalarial resistance.

Parasite genetic barcode extensions for modelling resistance

In order to model antimalarial resistance, we have adapted the parasite barcode used in Watson et al. for the simulation of resistance (Watson et al. 2021). In the new formulation, each position in the barcode represents either the absence (barcode position is equal to 0) or presence (barcode position is equal to 1) of a resistance mutation associated with resistance to a particular drug. For example, Supplementary Table 2 shows how a barcode with three loci can be used to represent resistance to DHA-PPQ and ASMQ.

Supplementary Table 2. Example barcode alterations to model antimalarial resistance

Artemisinin Resistance	Piperaquine Resistance	Mefloquine Resistance	Phenotype
0	0	0	Wild type parasite. Fully susceptible.
1	0	0	Resistant to artemisinin
0	1	0	Resistant to PPQ
0	0	1	Resistant to MQ
1	1	0	Resistant to DHA-PPQ
1	0	1	Resistant to ASMQ
0	1	1	Resistant to PPQ and MQ
1	1	1	Multidrug resistant to DHA-PPQ and ASMQ

Barcodes are used to track populations of parasites that are introduced from an infectious mosquito bite. We assume loci are genetically unlinked (genes known to confer resistance to the five first-line ACTs recommended by the WHO each occurring on different chromosomes) and consequently segregate independently during recombination.

Fitness Costs

Antimalarial resistance is assumed to introduce a fitness cost to resistant parasites compared to wild type parasites. Fitness costs are associated with each resistance locus and are assumed to be multiplicative. The fitness cost manifests as a reduction in parasite density that is assumed to reduce the probability of the resistant parasite being passed on to a mosquito. This can be expressed mathematically as follows. Let $b = \left[b_1, b_2, ..., b_m\right]$ describe the vector of barcode loci for a barcode of length m. For example, the ASMQ resistant strain in Supplementary Table 2 is represented by the vector $[1,\ 0,\ 1]$. If ν_j is the resistance cost associated with barcode locus j, then the comparative fitness cost due to resistance, r, for the given parasite is simply:

$$r = \prod_{i=j}^{m}(1 - b_j v_j)$$

Consequently, in this study the wild type allele at each locus is assumed to confer no fitness costs, with $(1 - b_j v_j)$ equal to 1 if locus j is wild-type, and thus $r = 1$ for the wild type parasite

in Supplementary Table 2. In this analysis, each resistance locus is assumed to have a fitness cost equal to 0.0005, i.e. $v_{i...m} = 0.0005$. As a result, the relative fitness of a genotype with k resistance mutations, in the absence of any drugs present in the blood, is $(1 - 0.005)^k$.

The number of oocysts generated from each mosquito bite on an infectious human is drawn from a zero-truncated negative binomial distribution with mean = 2.5 and shape = 1. The selection of which parasite strains from the infected individual contributed to the oocysts is given by the relative probability that a given strain will be chosen in an individual with n gametocytogenic parasite strains, and is given by:

$$cr = \left[c_1 r_1, \ c_2 r_2, ... c_n r_n\right]$$

r_i is the fitness cost associated with parasite strain i and c_i is the contribution of parasite i to onward infection, which will be either c_T, c_D, c_A or c_U depending on the infection status of strain i denoted here as X_i. As in the original model, c_T, c_D and c_U are constants, and c_A is dependent on an individual's immunity. The probability that an infected individual infects a mosquito is still determined by the set of parameters determining the onward contribution to transmission, $\left\{c_T, \ c_D, \ c_A, c_U\right\}$, which are based on the infection status and immunity of the individual, denoted here as Y. However, if the strains responsible for the human's current infection state, i.e. all strains that match the human's infection state, are resistant then the probability of onward transmission is determined by the highest onward contribution of these strains, which is given by:

$$max\left\{cr_i : X_i = Y\right\}$$

In this way, fitness costs both affect the relative probability that a resistant strain is transmitted compared to a wild type strain in a mixed infection, while also reducing the probability that transmission occurs in individuals where the highest parasite density strain is resistant.

Clinical and Treatment Outcomes

With the addition of resistance, the treatment efficacy now varies and is determined both by the genotype of the parasite strains, the parasite density of each strain and the drug used to treat

the infection. The probability that any given strain is cleared by drug z can be expressed as $ez_{\beta(b)}$, where $\beta(b)$ is an adapted conversion of binary to decimal integers and is given by:

$$\beta(b) = \left(\sum_{j=1}^{m} b_j . 2^{j-1}\right) - 1$$

Using the same representation of a parasite genotype as in Supplementary Table 2 the efficacy of drug z can be expressed by the vector $ez = \left[ez_1, ez_2, ..., ez_{2^m}\right]$ for simulations in which the number of loci being modelled is equal to m. ez_1 represents the efficacy of the drug against the wild type parasite, i.e. the barcode vector b represented by a vector of length m filled with zeros. Importantly, ez reflects the probability that the drug will clear a parasite that has led to a symptomatic infection, i.e. the parasite strain is at a sufficiently high parasite density to trigger symptoms and seek treatment. This is equivalent to the probability of successfully clearing a symptomatic infection such that the infection does not recrudesce and lead to a 28-day treatment failure.

In this analysis, we used the drug efficacy by genotype table parameterised in (Nguyen et al. 2021), which was used by each group to parameterise the efficacy of each drug on each resistance genotype. 64 genotypes are included by allowing for variation at the K76T locus in *pfcrt*, the N86Y and Y184F loci in *pfmdr1*, the C580Y locus in pfkelch13, copy-number variation (CNV) of *pfmdr1*, and CNV of the *plasmepsin-2,3* genes. CNV is only separated into ‘single copy’ or ‘multiple copies’. See Section 3 - Treatment efficacy on specific genotypes for more information about this. In the Imperial model, the efficacy of each drug determines the probability of a 28-day parasitological failure, with the probability of late parasitological failure for each parasite genotype modelled shown in Supplementary Table 3.

Parasites from previous infections are assumed to be at a lower parasite density than the infecting strain that triggered the clinical infection (and hence why the individual is seeking treatment) and will be more likely to be cleared by the drug. In the model, the infection state of each strain, X_i, as well as the day the strain was acquired, t_0, and the time the strain will move out of the infection state, t_1, is tracked. This information is used to define the probability that strain i will recrudesce after treatment, $P(Recrudesce)_i$, which is given by:

$$P(Recrudesce)_i = \begin{cases} ez_{\beta(b_i)}\ , & X_i = Y \\ ez_{\beta(b_i)}\left(\frac{t_1 - t_c}{t_1 - t_0}\right), & X_i = A \\ 0\ , & X_i = U \end{cases}$$

where t_c is the current time. $P(Recrudesce)_i$ assumes that parasites below ~200p/µl (state U) will always be cleared regardless of the parasite phenotype. $P(Recrudesce)_i$ also assumes that the probability that an asymptomatic parasite above ~200p/µl (state A) will recrudesce is linearly related to the age of the infection and is at its highest when it first enters state A.

The probability that a treated infection will be successfully cleared by drug z, $P(Cleared)$, is equal to one minus the highest probability of a strain recrudescing, which is given by:

$$P(Cleared) = 1 - max\{P(Recrudesce)_i : 0 \leq i \leq n\}$$

This assumes that the multiplicity of infection does not directly affect the probability that an individual will be cleared, i.e. only one Bernoulli trial with probability $P(Cleared)$ is used to determine if all parasite strains were cleared.

If an individual fails treatment, it is assumed that they will recrudesce to yield a late parasitological failure (LPF) and move into state A after the prophylactic period of the drug has finished. Whether each parasite strain in a multiply infected individual recrudesces during a LPF is dependent on $P(Recrudesce)_i$. Bernoulli trials are conducted for each parasite strain except for one random strain for which $P(Recrudesce)_i = P(Cleared)$, which ensures that one of the most likely strains to recrudesce did actually recrudesce and cause a LPF.

If an individual successfully clears all parasites, they will either move directly into a state P or they will remain in the treated compartment for a longer duration resulting from slow parasite clearance (SPC). SPC is assumed to always occur if any of the pre-treatment strains that contributed to the clinical disease were resistant to any component of the drug. The duration of SPC was set equal to 10 days based on previous modelling studies estimating parasite clearance rates associated with SPC (Slater et al. 2016). During SPC it is assumed that all

parasite strains not resistant to the drug given have cleared and will thus not contribute to onward infection during SPC.

Lastly, individuals in state P can be reinfected before returning to state S, depending on the genotype of the infecting strain, how recently they were treated and the ACT used, reflecting the different half-lives of available partner drugs. For all drugs the probability of reinfection in state P increases as the partner drug wanes. As described in (Bretscher et al. 2019), we use the gamma distribution, $\Gamma(\alpha_i, \beta_i)$, where α_i and β_i are the shape and rate parameter respectively of the Gamma distribution, for drug i, to describe the probability of reinfection in individuals treated with AL and ASAQ. We use $\alpha_{AL} = 93.5$ and $\alpha_{ASAQ} = 16.8$ for the shape parameters for AL and ASAQ. These values represent the mean posterior estimate for the shape parameter in (Bretscher et al. 2019), which were estimates across a number of trial sites in Africa. We use $\beta_{AL} = 5.22$ and $\beta_{ASAQ} = 0.94$ for the rate parameters, which yield mean durations of protection equal to 17.9 days and 17.8 days respectively. These durations are longer than the posterior mean duration of protection estimated in (Bretscher et al. 2019) and more closely reflect study sites in (Bretscher et al. 2019) with the longest times to reinfection for AL and ASAQ. This was chosen because the mean duration was estimated across study sites with known resistance markers and subsequently we chose to match to relationships in sites that were assumed to have the least amount of partner drug resistance for each drug. For DHA-PPQ, we assume the probability of reinfection is described by a Weibull survival curve, with scale and shape equal to 28.1 and 4.4 respectively, as estimated in (Okell et al. 2014) , with a mean duration of protection equal to 25 days. The described periods of prophylaxis are used to define the per-day probability of an individual being reinfected if the parasites introduced during an infectious bite are not resistant to the partner drug. However, if any of the introduced parasites are resistant to the partner drug, we assume a shorter period of prophylaxis. For AL and ASAQ, we use $\beta_{AL} = 10.75$ and $\beta_{ASAQ} = 1.45$, resulting in mean durations of protection equal to 8.7 days and 11.6 days respectively. These shorter durations were chosen to match to prophylactic profiles in sites in (Bretscher et al. 2019) with the shortest durations of protection due to the presence of partner drug resistance. For DHA-PPQ, we used a larger scale parameter of 59 for the Weibull survival curve, approximately halving the mean duration of protection to 12.4 days.

In order to simulate individual level variation in drug clearance and prophylaxis we first fit a Hill function to relate the assumed exponential clearance of the partner drug to the described period of prophylaxis for each drug on both wild type and resistant parasites. For each drug, we assume the mean drug lifetime is equal to $ln(2)$ * mean duration of prophylaxis of each drug against wild-type parasites (17.9, 17.8 and 25 days for AL, ASAQ and DHA-PPQ respectively from earlier prophylaxis fitting). When an individual moves from being treated to being in a state of prophylaxis, we draw the time at which they will become fully susceptible again from an exponential distribution with mean equal to the mean duration of prophylaxis for each drug. During this prophylactic period, we assume the individual's drug concentration decays exponentially such that they have fully eliminated the partner drug when returning to state S, with their drug concentration on each day used to calculate the probability of early reinfection using the earlier fitted Hill functions.

MORU Model

The base model has been extensively described in (Gao et al. 2020). Briefly, it is an individual based, discrete time, spatially explicit, stochastic model, with mosquito population dynamics as well as human population movement. The spatial features have been turned off for the purposes of the analyses presented here.

Population

As mentioned in the original model publication, since the vast majority of bites (at least for the vector species considered here) occur overnight, we are solely concerned with night time mosquito biting patterns and human behaviour. In general we would consider that daily population flows between villages are best characterised by a modified gravity model, but given that the models presented here were calibrated for different geographical settings and have different spatial resolutions and inherent human and mosquito mobility formulations, we chose to remove the human movement component of our model. Thus, to ensure that potential discrepancies between model predictions presented here are not driven by the significant differences in the mobility and spatial resolution components of the models, we assumed a closed population of 100,000 individuals, which in our framework is equivalent to discriminating a single village containing all humans and mosquitoes as described below.

Transmission

Transmission within a village follows a quasi-homogeneous process whereby each mosquito is equally likely to bite any individual. Given that people can move freely within their village whilst mosquitoes are actively seeking and biting, we can reasonably assume that all humans in a given village can potentially be bitten by any one mosquito in that village.

Malaria transmission is spatially heterogeneous (Erhart et al. 2005; Cui et al. 2012; Gryseels et al. 2015) as manifested by significantly different disease incidence rates within countries, but entomological data to inform the distribution of mosquito densities at scale is lacking. Due to this lack of mosquito abundance data and the fact that each model was calibrated for different settings where mosquito bionomics are likely to vary, and thus to minimise potential divergence across models, we assumed that humans and mosquitoes are enclosed in a single village of 100,000 individuals.

Immunity and Symptoms

Each human agent is assigned two properties in relation to immunity, namely Cumulative Number of Exposures (*cml*) and Immunity Level (*lvl*). Both properties are set to 0 for newborns. The likelihood of clinical symptoms brought on by a single infection is given by:

$$clinical_{prob_n} = exp(-\ 0.15(moi_n - 1))^{\frac{0.1exp(-0.1cml_n - 1)\ + exp(-0.9cml_n)}{lvl_n^{\ 0.5}}}$$

where *moi*, *cml* and *lvl* denote the multiplicity of infection, cumulative exposure to malaria, and immunity level properties of the human agent, respectively. Although we only increase the immunity level if individuals resolve their infection (presumably due to increased antibody killing activity), the cumulative exposure is updated with each infectious bite received. Thus, individuals can accrue some immunity with superinfections. One level of clinical immunity is gained by a human agent every time his infection list is emptied. Immunity loss starts 40 days after one level of immunity is gained. Immunity is lost at a rate of 60 days^{-1}. Therefore, each human agent at immune level one is clinically immune for an average of 100 days. A loss in immunity prompts a reduction in immunity level and not the immune status per se.

Non-clinical (asymptomatic) untreated infections are assumed to last an average of 160 days, following data from the malaria therapy experiments (Collins and Jeffery 1999).

Interventions

The base model includes a range of possible malaria control interventions, from Insecticide Treated Nets (ITNs/LLINs) to more extreme and logistically complex ones like Targeted Malaria Elimination (TME). For this model comparison exercise, we decided to remove all control interventions except baseline clinical malaria management. This is the foundation of any malaria control program as it can be parameterised to reflect the percentage of clinical infections that receive appropriate treatment (antimalarial drugs). The choice of drugs dispensed is made explicit in each of the presented results.

Genotype to phenotype map

In our base model, each infection is treated as an individual entity with its own life cycle within the blood system of a human agent, and its drug-resistance profile is a product of the genetic information in the six alleles conferring resistance to the drugs considered here. Genotype-specific drug efficacies are described in Section 3 - Treatment efficacy on specific genotypes.

Within host dynamics

PK/PD

We use a simple 1-compartment pharmacokinetic (PK) model that assumes drug concentration drops below a predefined minimum inhibitory concentration (MIC) following an exponentially distributed rate. The MIC defines the point in time after treatment at which the drugs administered are no longer able to counteract parasite growth, i.e. the day after which the infection can only be cleared by immunity. Our PK compartment does not reflect drug concentration, but rather a drug action status defining the potential parasite clearance by drug action. If the drug action status is ON, i.e. drug concentration is above the MIC, drug killing effects for each drug considered are implemented as a daily probability of clearance that results in the expected treatment failure rate at day 28 for each combination of drug given and parasite genotype. We assumed the mean times to reach MIC are 25 days for amodiaquine, 14 days for lumefantrine, and 30 days for piperaquine. Artemisinin-derivatives are assumed to only be present at the day of dosing.

Parasite killing rates depend on the person's transmission status (*s*), with parasite clearance in not yet infectious people generally slower than that in individuals carrying gametocytes. Clearance of parasites with drug resistance phenotype *h* by drug *d* then follows:

$$Clearance_{shd} \sim B(N_{shd}, c_{shd})$$

where c_{shd} is an element of a 3-dimensional matrix *C* of size |S|*|H|*|D| and *B* denotes a binomial distribution. N_{shd} determines the number of parasite sub-populations affected by c_{shd},

thus a human host is only declared cleared from infection once every parasite sub-population has been cleared from their bloodstream. The parameter values in c_{shd} have been calibrated to produce the expected treatment failure rates in Section 3, as per the common genotype-specific drug efficacies table, given the drug PK described above.

Mutation

Per parasite subpopulation, we consider a daily probability of mutation at any of the alleles conferring resistance to either artemisinin or the partner drugs. These mutations are considered independent events meaning they may happen on separate days or on the same day, and there is no precedence between the two events. A base mutation rate of 10^{-9} is used in the absence of drug pressure. That rate is five orders of magnitude higher when drugs are present in the blood system (Pongtavornpinyo et al. 2009). Note that the "mutation rate" here (as in all models that do not have explicit classes for within-host parasite dynamics) refers to both the probability of mutation and the probability of within-host fixation of the new mutant. Probability of within-host fixation is higher under drug pressure than in the absence of drug pressure.

Clone selection

Our base model allows for co-infection with a large number of clones (defined as parasite populations originating from different inocula, regardless of genotype). In the version used here, we simplify within-host dynamics by imposing a set of rules to determine the likelihood of fixation of different parasite clones under different drug pressure contexts. Given the assumed differences in parasite fitness according to genotype, we determine that: *i*) in the presence of combination therapy, double resistant parasites are likely to fix if present, followed by single resistant genotypes. Thus, if the parasite population is a mix of single mutants and wild-type parasites, the single mutant parasites will eventually outcompete the wild-type parasites under drug pressure, will form the bulk of mature gametocytes, and subsequently be transmitted to mosquitoes upon a bite. If there is a mix of double mutants with any other genotype subpopulation, the double mutant population will fix. *ii*) when there are no drugs in the bloodstream, a random draw decides which parasite subpopulation will be transmitted to the mosquito. This effect tries to account for infections where the drug-resistance allele emerges at

different infection time points, infections where drugs were given at subtherapeutic levels, and infections with a greater multiplicity of infection, and is re-evaluated over the course of the infection, upon a new mosquito bite. Importantly, transmissibility of different clones to mosquitoes is obviously dependent on relative parasite frequencies of different clones, which are a direct result of the dynamic processes described above. Using an expanded version of an in-house within-host *P. falciparum* model (Saralamba et al. 2011), we determined that there is a critical time window for the fixation of resistant parasite populations (Supplementary Figure 14) Independently of when the resistance clones emerge (middle panel), fixation seems to always occur at day 5-7 (right-side panel) post drug treatment. This finding allowed us to set a six-day lag for the higher mutation rate to be applied. This means that for the first six days after treatment is initiated, the base mutation rate is used; if after six days there is still drug present in the blood, the higher mutation rate is applied. Once there is no drug left in the blood, the mutation rate reverts to the base rate if the infection has not yet been cleared. We thus are able to reproduce the complex within host dynamics generated by far more complex models by modulating the use of the mutation rates and asserting fixed allelic dominance functions that determine which parasite clones will fix under different drug pressure scenarios. We do not model back mutation events.

PSU Model

Model characteristics are described in detail in (Nguyen et al. 2015, 2021), and summarized below with changes noted.

Population

A population of 100,000 individuals is modelled. Individuals can be uninfected, or infected with one or more clones of *Plasmodium falciparum*; each clone is its own genotype. In low-transmission regions, the vast majority of infections are single-clonal, while in high-transmission regions mult-clonal infections are common. The model's distribution of clones per person is calibrated to data in (Owusu-Agyei et al. 2002) from a high-transmission region of northern Ghana. Mosquito bites occur on individual hosts in the model determined by each person's 'biting attractiveness' parameter. Biting attractiveness is assigned at birth in the model, and is drawn from a gamma distribution with a coefficient of variation of 2.0 (Smith et al. 2005). An extrinsic incubation period of 11 days is modelled, and new infections are generated every day in the model based on genotypes that were sampled by mosquitoes 11 days ago. Genotype distributions drawn 11 days ago are adjusted to account for recombination occurring in hosts with multi-clonal infections, with a standard recombination table. In other words, a 64 x 64 x 64 table is maintained showing the probability that genotype A recombining with genotype B would form genotype C. The within-host frequencies of genotypes A and B give the probability of sampling both genotype A and B, and the value in the table gives the probability that the offspring is genotype C. A final genotype distribution -- based on the frequencies of currently circulating genotypes, and the probabilities that their offspring after recombination are "genotype C" -- is used to determine onward infection to new hosts.

Parasitaemia and Immunity

Each clone in each host has its own blood-stage parasite density variable which influences transmission probability to mosquitoes, according to (Ross, Killeen, and Smith 2006). Gametocyte density is assumed to be proportional to asexual density, and gametocyte dynamics are not modeled separately; however a 'delayed version' of the asexual-density variable is used to determine onward transmission as rises and falls in gametocyte densities

mostly follow those in asexual densities. Parasitaemia in hosts goes up after an infectious bite that results in symptoms, with peak parasite density at the point of fever/presentation drawn randomly to be between 2000 parasite/μl and 200,000 parasite/μl (uniform distribution on $\log_{10}$-scale, between 3.3 and 5.3). An infectious bite that does not result in symptoms will start with a mean parasitaemia given by 10^x, where x is drawn from a normal distribution with mean 3 and standard deviation 0.5. This gives a mean starting parasitaemia level of 1000/μl with 95% of draws giving a parasite density between 100/μl and 10,000/μl for asymptomatic individuals.

Duration of Infection

Asymptomatic infections are cleared according to a host's immune level *M*, which is simply a relative indicator between 0.0 and 1.0 of minimum and maximum clearance rates by the immune system. Daily clearance rates by the immune system occur at the rate:

$$D_{t+1} = w_R \cdot [0.8036(1 - M_t) + 0.9572 M_t] \cdot D_t$$

and are calibrated (as explained in supplement section 1 of (Nguyen et al. 2015)) to several other well-known data sets and approaches ((Filipe et al. 2007; Molineaux and Gramiccia 1980; Maire et al. 2006; Eyles and Young 1951)). Infections are a minimum of 60 days long and a maximum of 281 days long.

Symptomatic Infection

Upon an infectious bite, a host will progress to a case of symptomatic febrile malaria with probability

$$P_{clin} = \frac{0.99}{1 + (M / 0.4)^4}$$

and with probability 1 - P_{clin} the parasites from the new bite will expand to a parasite density between 100/μl and 10,000/μl and establish as an asymptomatic infection. M in the above equation refers to the host's current level of immunity. This calibration was done to several clinical-incidence-by-age data sets from Africa (section 11 of supplement, (Nguyen et al. 2015))

PK/PD

A 1-compartment pharmacokinetic (PK) model is used with basic exponential decay modelling drug clearance. Half-lives used are 9 days for amodiaquine, 4.5 days for lumefantrine, and 28 days for piperaquine. Half-lives for artemisinin derivatives are not modelled as they are very short-lived and the simulation has a one-day time-step. Artemisinin-derivatives are simply "present" in the blood on the day of dosing, absent the day after dosing (if no other dose is taken), and are modelled to have a daily p_{max} = 0.999 on drug-sensitive parasites, which translates to a daily killing rate of p=0.9982 for an average drug concentration (C=1.0). Daily dose for patients is allowed to vary between patients and between dosing days. The day-1 artemisinin concentration C for a patient is drawn from a Normal(1.0, σ=0.4) and subsequent dosing days (i.e. days 2 and 3) can have C adjusted up or down (relative to day 1) by a draw from Uniform(-0.2,0.2). When C=0.9, p=0.9886. When C=0.8, p=0.8331. These numbers were calibrated in Nguyen et al (2021) to obtain a 3-day artesunate monotherapy efficacy of 68.9%.

Fitness costs

All single-resistant parasites are assumed to have a daily fitness cost c_1 = 0.0005, which translates to an annual fitness cost of 0.167, i.e. a relative fitness of 0.833. Each resistance mutation (i.e. non-wild-type) adds an additional and equal fitness cost, and fitness costs are multiplicative and independent. In other words, the relative fitness of a genotype with k resistance mutations, in the absence of any drugs present in the blood, is $(1 - c_1)^k$.

3 Treatment efficacy on specific genotypes

All three models (PSU, MORU, Imperial) use the same locus-based drug-resistance model and genotype-specific drug efficacies. 64 genotypes are included by allowing for variation at the K76T locus in *pfcrt*, the N86Y and Y184F loci in *pfmdr1*, the C580Y locus in pfkelch13, copy-number variation (CNV) of *pfmdr1*, and CNV of the *plasmepsin-2,3* genes. CNV is only separated into ‘single copy’ or ‘multiple copies’. With 4 drugs used in the simulation -- artemisinin derivatives, lumefantrine, amodiaquine, piperaquine -- a total 64 x 4 = 256 drug-genotype interactions need to be modelled as an individual can be infected with any genotype and treated with any combination therapy. The monotherapy parameters need to be input into the simulation as well in case an individual is bitten and infected during a time window when only one residual drug is present in the blood. Each of the 256 drug-genotype combinations is parameterized by a p_{max} value (maximum killing rate) and an EC50 value (concentration at which killing power is reduced by half). These values are calibrated in a single-compartment PK/PD model to obtain efficacies that match the clinical trial literature, with some inference and imputation on what genotypes would have been circulating at the time each clinical trial was run. In the table below, a total of 64 x 3 = 192 efficacies are presented for three ACTs and 64 genotypes. See Supplementary Appendix 2 in (Nguyen et al. 2021) for full details as well as 28-day on calculation of treatment efficacies of each ACT modelled against each genotype in Supplementary Table 3.

In Supplementary Table 3 (below), the 6-character genotype code (e.g. KNY1C1) is, from left to right: K76T, N86Y, Y184F, CNV of pfmdr1, C580Y, CNV of plasmepsin-2,3 genes. To highlight the contribution of specific alleles towards treatment efficacies, we constructed a random forest model (Breiman 2001) using all the data in the drug by genotype table (i.e. no test/train split or cross validation as we were focussed on a maximally predictive model for our dataset only) to predict treatment efficacy based on the presence/absence of specific alleles. The importance of each allele in the random forest based on the mean decrease in the Gini index is shown in Supplementary Figure 15 with the predictions of the random forest. The overall performance of the random forest models is summarised using the mean absolute error, root mean squared error and the correlation (R^2), also shown in Supplementary Figure 15.

Supplementary Table 3: ACT 28-day probability of parasite clearance

Genotype	DHAPPQ	ASAQ	AL
KNY1C1	0.972486	0.962073	0.915158
TNY1C1	0.972486	0.947158	0.929358
KYY1C1	0.972486	0.905281	0.953453
TYY1C1	0.972486	0.891124	0.964582
KNF1C1	0.972486	0.976982	0.889945
TNF1C1	0.972486	0.958879	0.908054
KYF1C1	0.972486	0.930465	0.915158
TYF1C1	0.972486	0.917383	0.929358
KNY2C1	0.972486	0.962073	0.858645
TNY2C1	0.972486	0.947158	0.869483
KYY2C1	0.972486	0.905281	0.896817
TYY2C1	0.972486	0.891124	0.915158
KNF2C1	0.972486	0.976982	0.830015
TNF2C1	0.972486	0.958879	0.844322
KYF2C1	0.972486	0.930465	0.858645
TYF2C1	0.972486	0.917383	0.869483
KNY1Y1	0.928664	0.895737	0.795336
TNY1Y1	0.928664	0.864359	0.828863
KYY1Y1	0.928664	0.771709	0.877727
TYY1Y1	0.928664	0.735268	0.907645
KNF1Y1	0.928664	0.948359	0.723351
TNF1Y1	0.928664	0.892625	0.753201
KYF1Y1	0.928664	0.824725	0.795336
TYF1Y1	0.928664	0.794297	0.828863
KNY2Y1	0.928664	0.895737	0.646288
TNY2Y1	0.928664	0.864359	0.684442
KYY2Y1	0.928664	0.771709	0.75155
TYY2Y1	0.928664	0.735268	0.795336
KNF2Y1	0.928664	0.948359	0.570356
TNF2Y1	0.928664	0.892625	0.604481
KYF2Y1	0.928664	0.824725	0.646288
TYF2Y1	0.928664	0.794297	0.684442
KNY1C2	0.768484	0.962073	0.915158
TNY1C2	0.768484	0.947158	0.929358
KYY1C2	0.768484	0.905281	0.953453
TYY1C2	0.768484	0.891124	0.964582
KNF1C2	0.768484	0.976982	0.889945
TNF1C2	0.768484	0.958879	0.908054
KYF1C2	0.768484	0.930465	0.915158
TYF1C2	0.768484	0.917383	0.929358
KNY2C2	0.768484	0.962073	0.858645
TNY2C2	0.768484	0.947158	0.869483
KYY2C2	0.768484	0.905281	0.896817
TYY2C2	0.768484	0.891124	0.915158
KNF2C2	0.768484	0.976982	0.830015
TNF2C2	0.768484	0.958879	0.844322
KYF2C2	0.768484	0.930465	0.858645
TYF2C2	0.768484	0.917383	0.869483
KNY1Y2	0.414973	0.895737	0.795336
TNY1Y2	0.414973	0.864359	0.828863
KYY1Y2	0.414973	0.771709	0.877727
TYY1Y2	0.414973	0.735268	0.907645
KNF1Y2	0.414973	0.948359	0.723351
TNF1Y2	0.414973	0.892625	0.753201
KYF1Y2	0.414973	0.824725	0.795336
TYF1Y2	0.414973	0.794297	0.828863
KNY2Y2	0.414973	0.895737	0.646288
TNY2Y2	0.414973	0.864359	0.684442
KYY2Y2	0.414973	0.771709	0.75155
TYY2Y2	0.414973	0.735268	0.795336
KNF2Y2	0.414973	0.948359	0.570356
TNF2Y2	0.414973	0.892625	0.604481
KYF2Y2	0.414973	0.824725	0.646288
TYF2Y2	0.414973	0.794297	0.684442

4 Sensitivity Analyses and Outcome Measures

The main analysis consisted of simulating 100 realisations of 100,000 individuals living in a particular transmission setting (PfPR=1%, 5%, 10%, 20%), with varying access to antimalarial drugs if febrile (coverage=20%, 40%, 60%). One primary ACT was used as first-line therapy (DHA-PPQ, ASAQ, AL) and five different allele frequencies of pre-existing partner drug resistance (0.0, 0.01, 0.10, 0.25, 0.50) at time = 0 were explored, resulting in 18,000 simulations by each model. For each scenario, models are run until a steady-state prevalence is achieved prior to evaluating resistance evolution over a 40-year period. For our sensitivity analyses, we chose one transmission setting (PfPR = 20%) and one treatment coverage level (40%) and conducted 50 realisations of 100,000 individuals, with each sensitivity analysis scanning across seven parameter values (yielding 10,500 simulations per sensitivity analysis by each model) to characterise the impact of the following drivers of resistance.

4.1 Resistant genotypes' fitness costs

The default daily fitness cost of resistance, c, was assumed to be equal to 0.0005. The fitness of the wild-type parasite KNY1C1 is defined as 1.0, and the fitness of a resistant strain is defined as $1.0 - c$. In the sensitivity analysis, we explored increasing and decreasing c by 10%, 20% and 30%. If a model has multiple fitness costs (for example affecting within-host parasite densities and/or onward probability of transmission) all fitness costs will be increased or reduced by the same amount.

4.2 Genotype-specific drug efficacies

To explore the impact of drug efficacies, we altered our assumptions about the default efficacy of each ACT on each parasite genotype. We explored increasing and decreasing the efficacy of each drug by 15%, 10% and 5%. In the PSU model, this was modelled by varying the EC50 values by 5%, 10%, and 15%, generating 6 new drug efficacy by genotype tables. These tables were used directly by the Imperial model giving altered probabilities of 28-day treatment failure. Lastly, the MORU model varied the killing rate by 5%, 10% and 15%.

4.3 Duration of infection of asymptomatic carriage

To characterise the role of asymptomatic infections, we varied the duration of asymptomatic infections in the third sensitivity analysis. Asymptomatic infections, resulting from both drug treatment failure and infections that did not result in a clinical case of infection, were increased or decreased by 10%, 20%, 30%. In the Imperial model this was included by altering the duration of asymptomatic infections, while leaving the duration of the following sub-patent infection the same. In the MORU model, the mean duration of an untreated malaria infection was altered.

4.4 Probability of progressing to symptoms after an infectious bite

The rate at which new infections seek treatment will affect the selective pressure on antimalarial resistance. To explore this, we increased or decreased the probability that an individual will develop a clinical infection, i.e. an infection that is sufficiently severe in order to require treatment, by 5%, 10% and 15%. These changes alter only the probability of developing symptoms and not the probability that an individual will seek treatment, which remains fixed at 40% (treatment coverage). In all models, this change was implemented by altering both the maximum and minimum probability of developing symptoms dependent on the individual's immunity.

As in the main analysis, the main reported metric is the allele frequency of 580Y (a proxy for artemisinin resistance). Allele frequency was calculated as the weighted number of parasite-positive individuals carrying the 580Y allele / total number of parasite-positive individuals. The weights for each person describe the fraction of their clonal populations carrying 580Y, e.g. an individual host with five clonal infections two of which carry 580Y would be given a weight of 0.4.

5 Impact of altering relationship between genotypes and drug efficacy

The analyses presented throughout have assumed the same relationship between parasite genotype and drug treatment efficacy, i.e. we have used the same drug by genotype efficacy table (Supplementary Table 3). The individual values in this table are sourced from (Nguyen et al. 2021), which sourced estimates where available from previous drug efficacy studies with known parasite genotypes. In this analysis, model based inference had to also be leveraged to infer a number of the individual values for which no drug efficacy study was found in the literature, which may result in a number of the values in the drug by genotype efficacy table being off and not representing the true value. In response, we conducted a separate set of analyses to quantify the impact of uncertainty in the relationships between the modelled drug by genotype efficacies.

5.1 Uncertainty in estimates of drug efficacy by genotype

We generated 10 additional DxG tables by randomly drawing new EC50 values for each drug component from a normal distribution with mean equal to the default EC50 value and standard deviation = 0.1, i.e. 10%. The resultant 10 drug by genotype efficacy tables are shown in Supplementary Figure 16. Each drug by efficacy table was subsequently used to conduct the following simulations (Supplementary Figure 17), reporting as before the summary times to 0.25 580Y:

- 10% PfPR
- 40% treatment coverage
- 5 pre-existing partner drug frequencies (0, 0.01, 0.1, 025, 0.5)
- AL, ASAQ, DHAPPQ each explored
- 20 simulation repetitions per setting

5.2 Comparable drug efficacy by genotype across ACT

The simulations explored above (5.1 Uncertainty in estimates of drug efficacy by genotype) were chosen to explore the impact of uncertainty in drug efficacies to be explored. We also explored an alternative approach to characterise what impact the genetic landscape, i.e. the different mutational routes for maximum resistance to form, has on selection speed. To achieve this, we scaled the EC50 of lumefantrine and amodiaquine such that the most resistant genotype to AL and ASAQ had comparable treatment failure to the most resistant DHA-PPQ

genotype. In this way, the genetic relationship between different combinations of genotypes was maintained but the overall treatment efficacy decreased. This resulted in one new drug by genotype efficacy table (Supplementary Figure 18), which was used to explore the order in which 580Y was selected, i.e. whether 580Y or partner drug resistance was selected first (Supplementary Figure 19).

Lastly, this analysis also allowed us to interrogate further which model components were driving the difference in emergence times between the models, notably the faster emergence time in the PSU model (Figure 2). In Supplementary Figure 19, selection of partner drug resistance in the PSU model was observed to be quicker than selection of other resistance genotypes in other drug scenarios that had comparable treatment efficacy (e.g. the KNY0Y1 genotype in the AL scenario). In this analysis, selection was proportional to both the treatment efficacy and the half life of the partner drug, explaining why in the PSU model selection for PPQ resistance occurred at a rate quicker than for 580Y in the AL scenario. This model feature is specific to the PSU model, with the Imperial model displaying comparable selection speeds for PPQ resistance in the DHA-PPQ scenario and for the KNY0Y1 genotype in the AL scenario.

Supplementary Figures

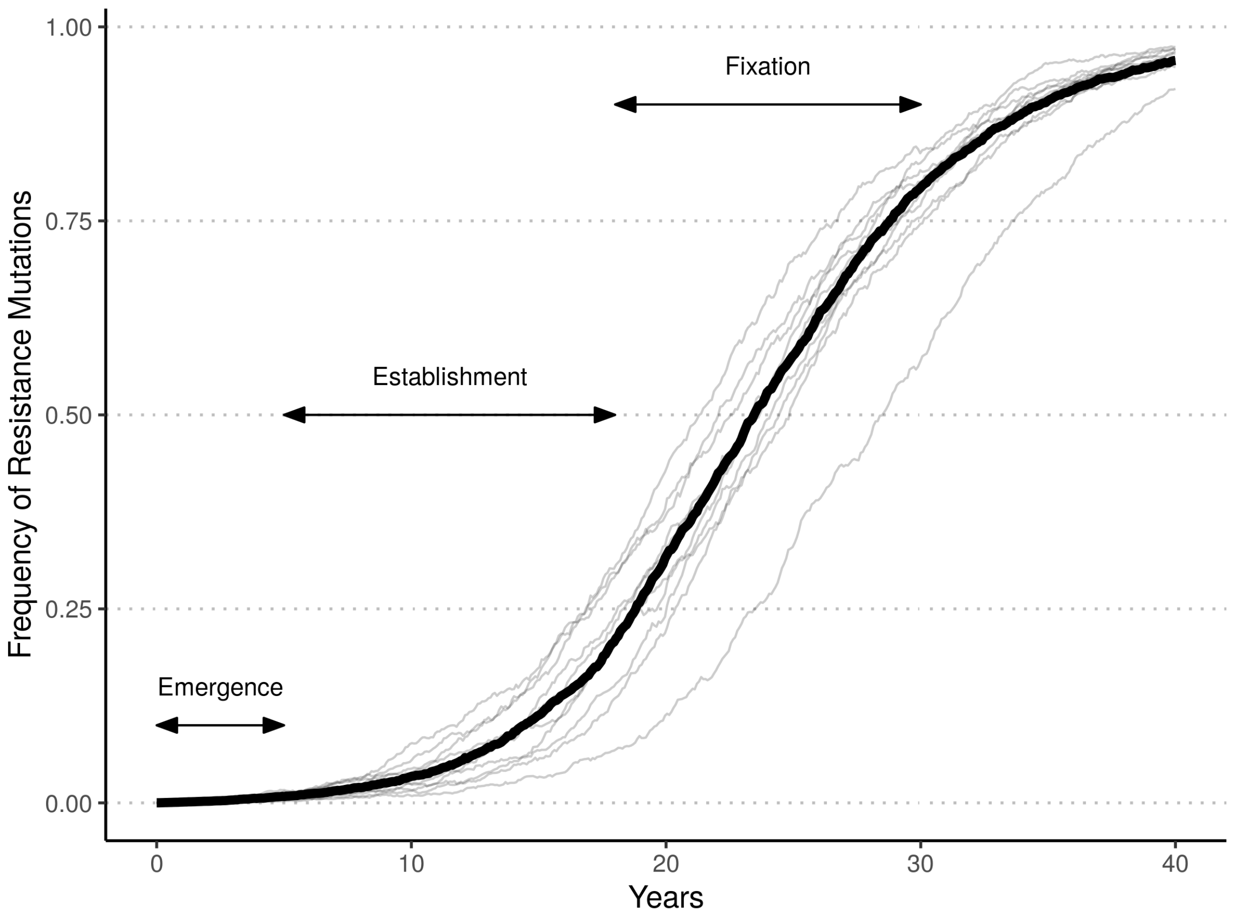

Supplementary Figure 1. Stages of drug resistance development. Drug resistance development is often typified by three phases describing the speed at which resistance increases; Emergence, Establishment and Fixation. Prior to emergence, mutations must first arise that confer drug resistance. Due to the rarity of these events and the low effective population size of mutant strains during emergence, this stage may vary in its length due to stochastic effects. After mutant strains have persisted through a number of transmission cycles, the spread of resistance occurs leading to the rapid increase in mutation frequency during establishment, during which stochastic effects are minimised. Following establishment, the increase in resistance mutations occurs approximately linearly at the beginning of fixation, before slowing until they reach fixation. An example of ten stochastic repetitions from our study for one scenario are shown with the median resistance frequency increase shown in bold. The timing of emergence (frequency 0 - 0.01), establishment (frequency 0.01 - 0.25) and fixation (frequency 0.25 - 1.00) are shown with two headed arrows.

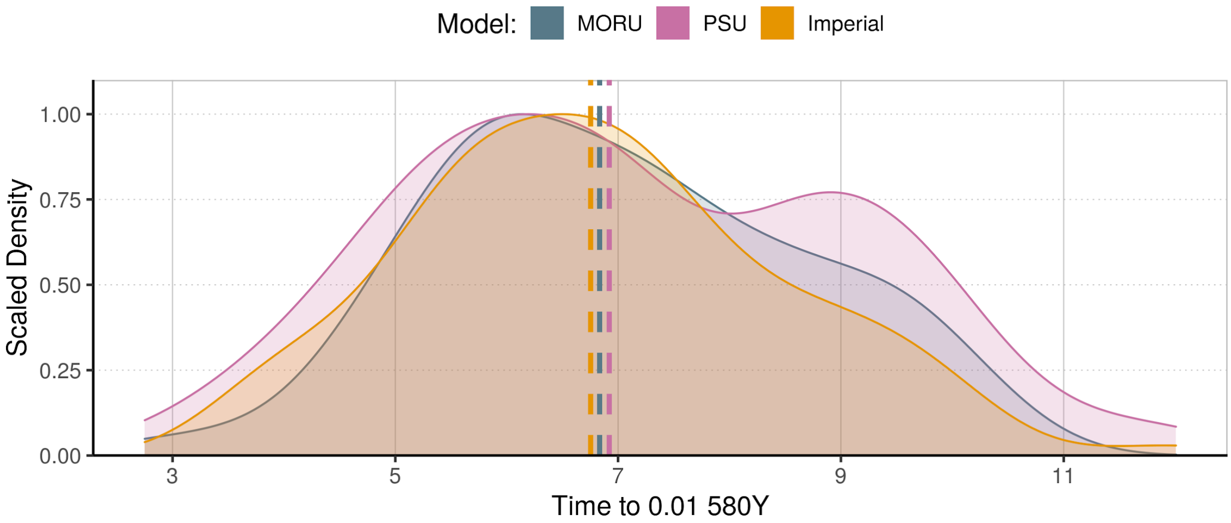

Supplementary Figure 2. Alignment exercise for 7 years till 0.01 580Y frequency. Scaled density for model times to 0.01 frequency of 580Y as part of model alignment exercise. We aligned the three models' de novo mutation rates forcing the models to reach 0.01 allele frequency of 580Y (i.e. early artemisinin resistance emergence) after seven years exactly under a specified set of conditions: 100,000 individuals in a transmission setting with all-ages PfPR=10% and 40% coverage with DHA-PPQ as a first-line therapy.

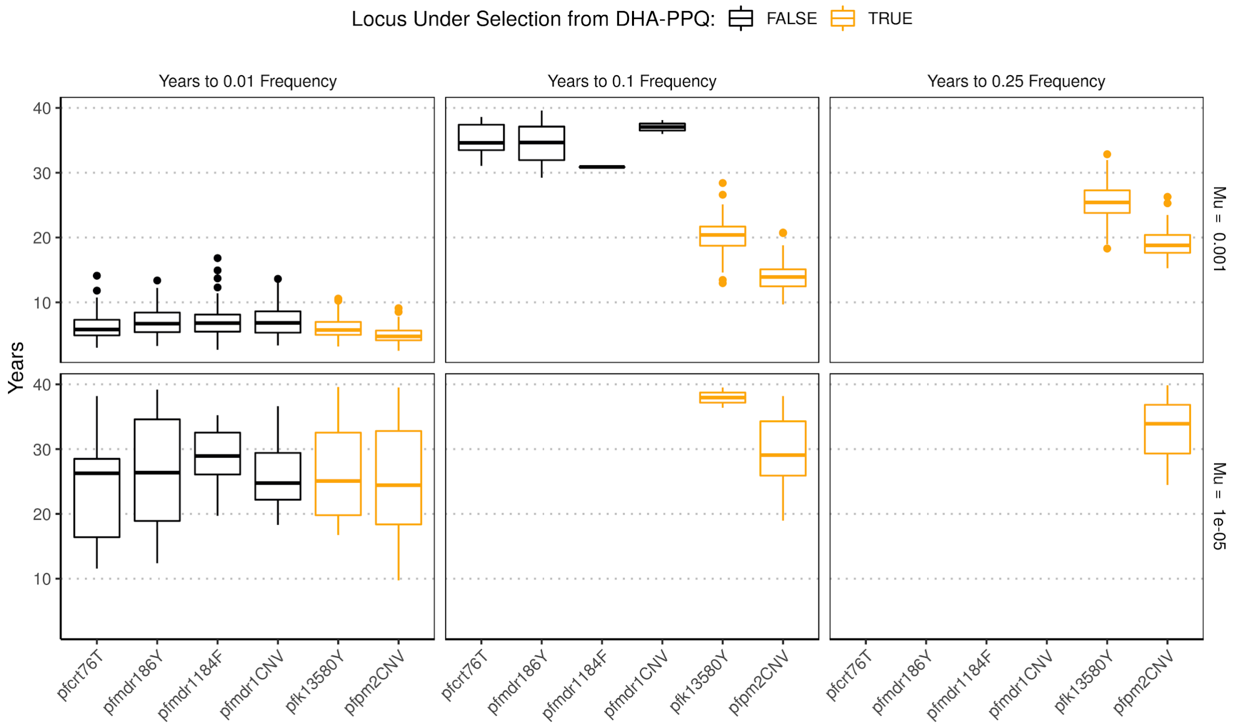

Supplementary Figure 3: Demonstration of equivalent emergence times for artemisinin resistance and loci not under selection. Distribution of times till different drug resistance frequency milestones (0.01, 0.1, 0.25 frequency) for each locus considered in a scenario in which DHA-PPQ is the first-line therapy. All clones are the wild-type genotype at the beginning of the simulation, with the same mutation rate (shown in rows) assumed for each locus. The two loci under selection by DHA-PPQ (pfk13-580Y and pfpm2-CNV) are shown in gold and the other loci, which confer no fitness cost or resistance advantage in the scenarios considered, are shown in black. An approximately equivalent time until 0.01 resistance frequency is observed for all loci, indicating that the time to emergence (0.01) is driven largely by the mutation rate chosen rather than the impacts of selection on arising mutations. For larger drug resistance milestones (0.1, 0.25) the resistance phenotype associated with pfk13-580Y and pfpm2-CNV results in shorter times till 0.1 and 0.25 frequency, indicating that these times are driven by the resistance phenotype rather than the mutation rate chosen.

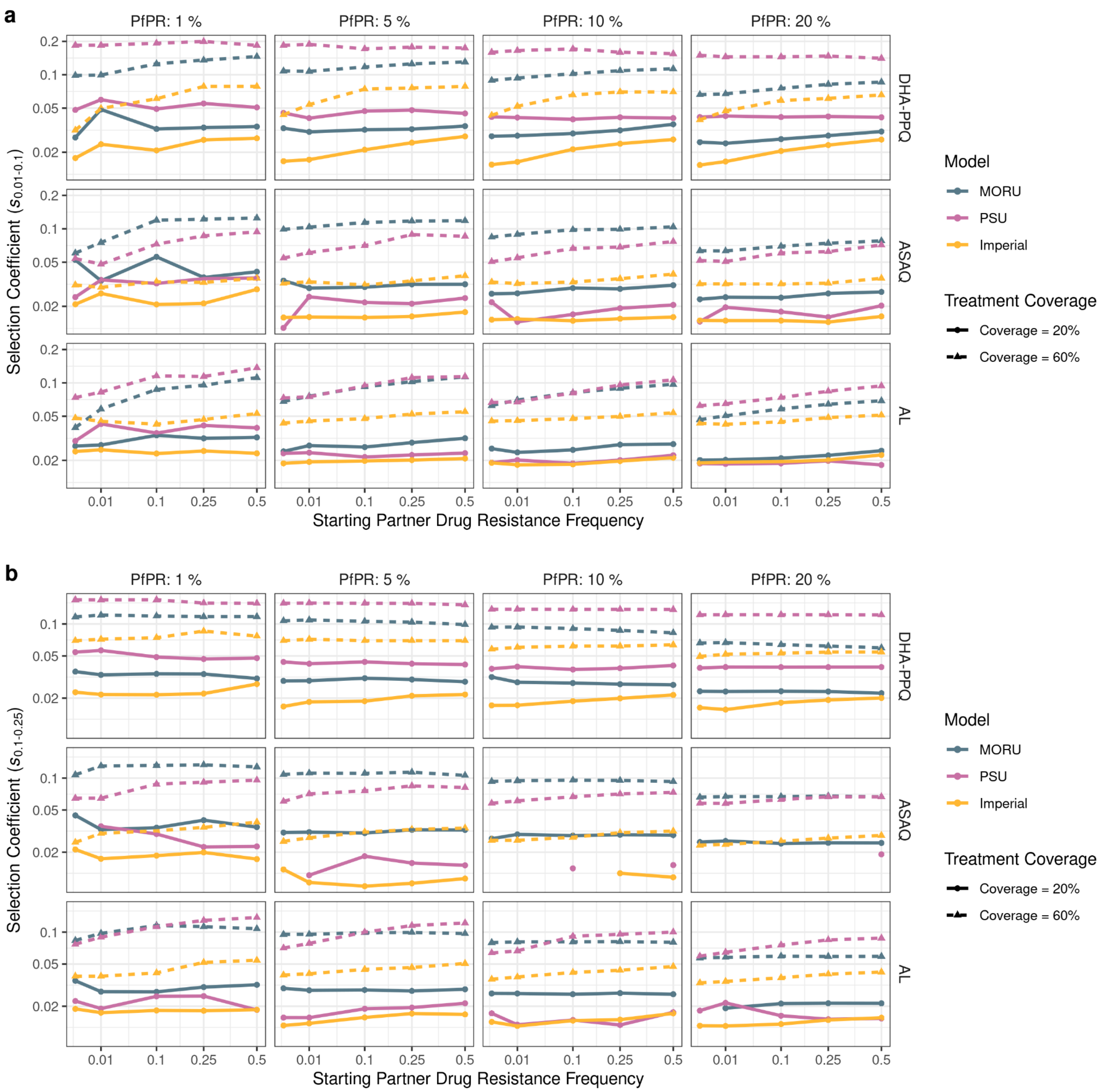

Supplementary Figure 4: Impact of starting partner drug resistance frequency on selection coefficients of 580Y. Median selection coefficients are shown, calculated using time taken for artemisinin resistance to increase from **a)** 1% to 10% ($s_{0.01-0.1}$) and **b)** 10% to 25% ($s_{0.1-0.25}$). Line type and shape of point show the assumed treatment coverage and the colour indicates the different transmission models used. Each column shows a different parasite prevalence and each row is a different assumed first-line ACT. For the simplest drug resistance mechanism (DHA-PPQ) the selection coefficients are notably flatter with respect to partner drug resistance in **b)** reflecting the constant rate of selection once resistance has reached the exponential stage of growth and is unaffected by stochastic fluctuations during the emergence of resistance. These dynamics are less pronounced for AL and ASAQ, which display increased selection coefficients with increasing partner drug resistance.

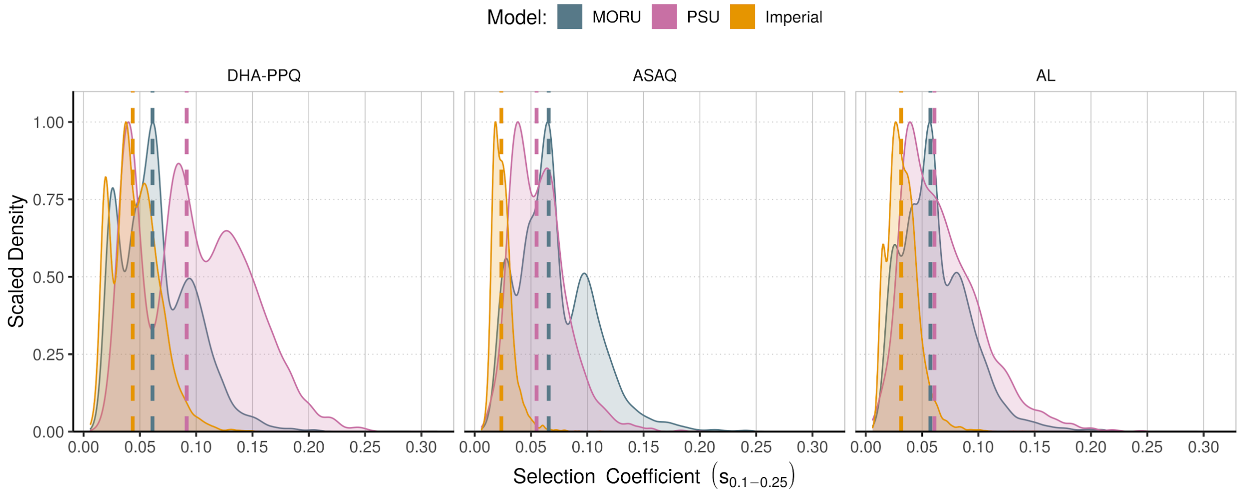

Supplementary Figure 5: Comparison between model selection coefficients. The selection coefficients are shown for each model and first-line drug, calculated using time taken for artemisinin resistance to increase from 10% to 25% ($s_{0.1-0.25}$). The scaled density shown reflects the overall selection coefficient across all scenarios considered (malaria prevalence, starting partner drug resistance and treatment coverage), with the median selection indicated with a vertical dashed line.

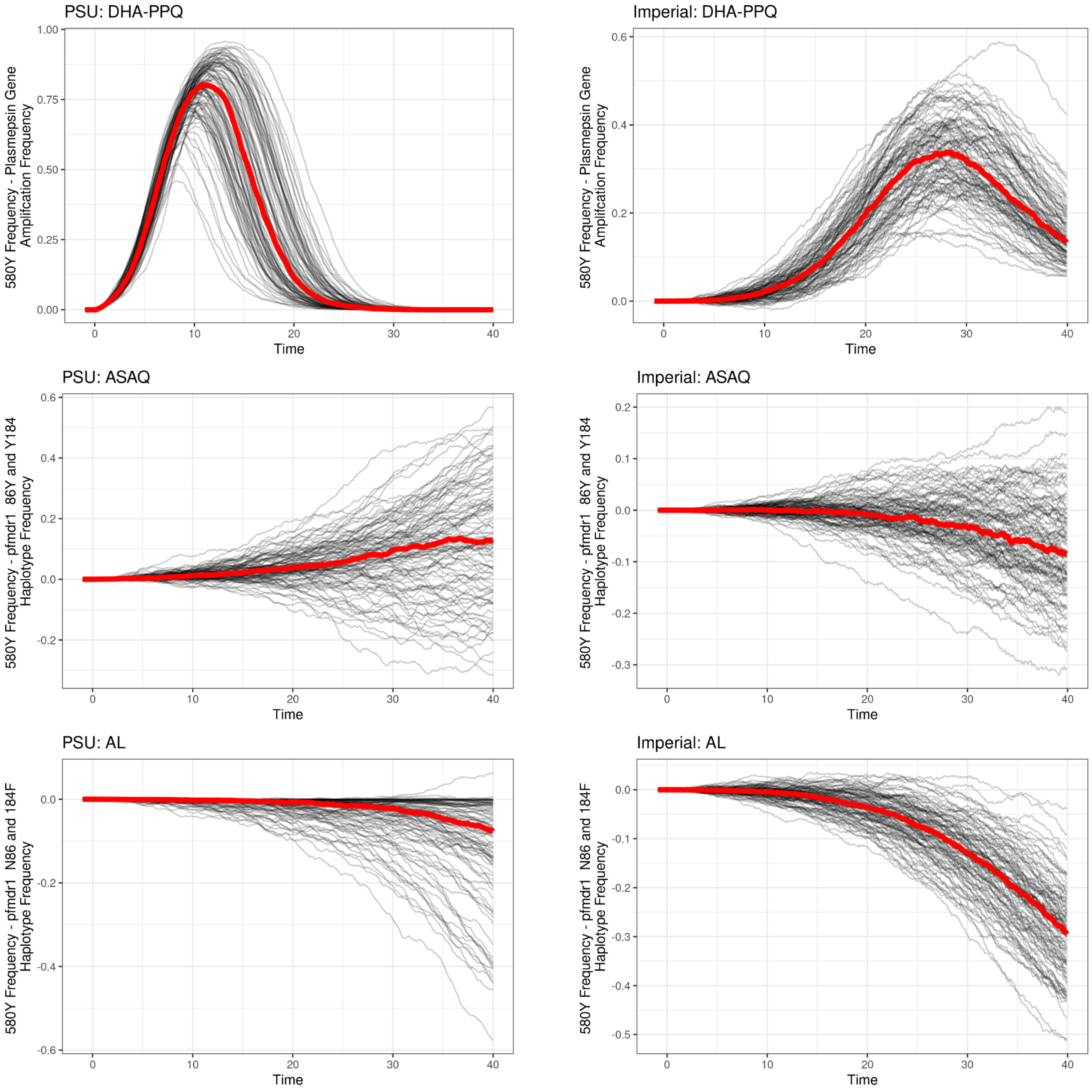

Supplementary Figure 6: Mutational Pathways towards multidrug resistance. The difference between 580Y and partner drug resistance genotype frequencies is shown over time for scenarios with no starting partner drug resistance, 10% PfPR and 40% treatment coverage. Positive values indicate that the partner drug resistance emerged quickest, whereas negative values suggest that 580Y emerges faster. In DHA-PPQ, partner drug resistance emerges before 580Y, with the trajectory of each simulation returning to 0 towards the end of the 40 year period, indicating that 580Y is being selected on a background of PPQ resistance. For AL, the opposite pattern emerges, with 580Y mutations being selected first. Lastly, for ASAQ, it appears that either mutation route is likely (partner drug or artemisinin resistance first). Each plot shows 100 simulation realisations, with the median trajectory shown in red.

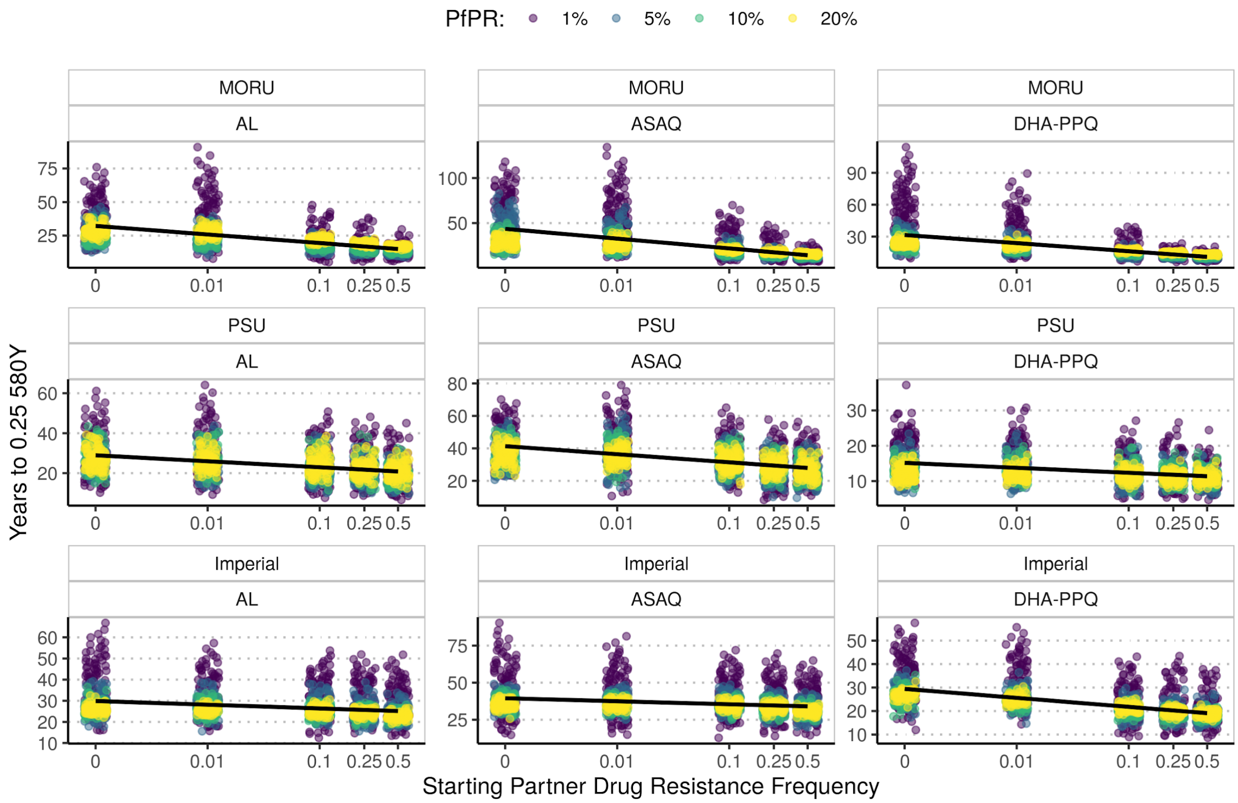

Supplementary Figure 7: Time (years) to artemisinin resistance establishment. The time for 580Y to reach 0.25 frequency is shown for each model and first line therapy. The points shown are jittered to ease representation of the variance in times observed for each starting partner drug resistance frequency (0, 0.01, 0.1, 0.25, 0.5). The parasite prevalence (PfPR) is shown by the colour of the points revealing greater variance in emergence times at lower parasite prevalence. The approximate log linear relationship is indicated by the black line. For times greater than 40 years, a survival model was used to generate the times till 0.25 580Y.

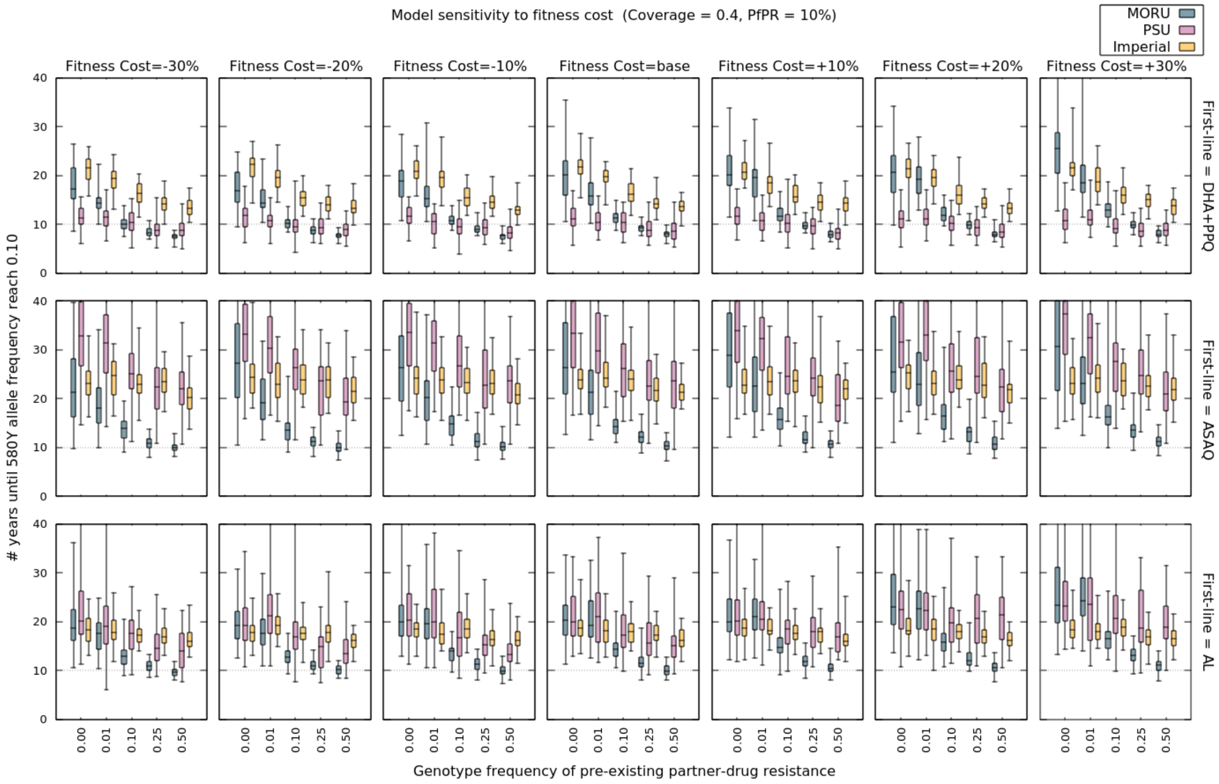

Supplementary Figure 8: Number or years until 580Y allele frequency to increase from 0.00 to 0.25 in regions depending on assumed fitness cost of resistance. The box (median and interquartile range) and whisker (95% quantile range from 50 simulation realisations) plots presented are censored boxplots, with simulations only being run for 40 years. Values greater than 40 years contribute to the median, quartiles and 95% ranges if calculable.

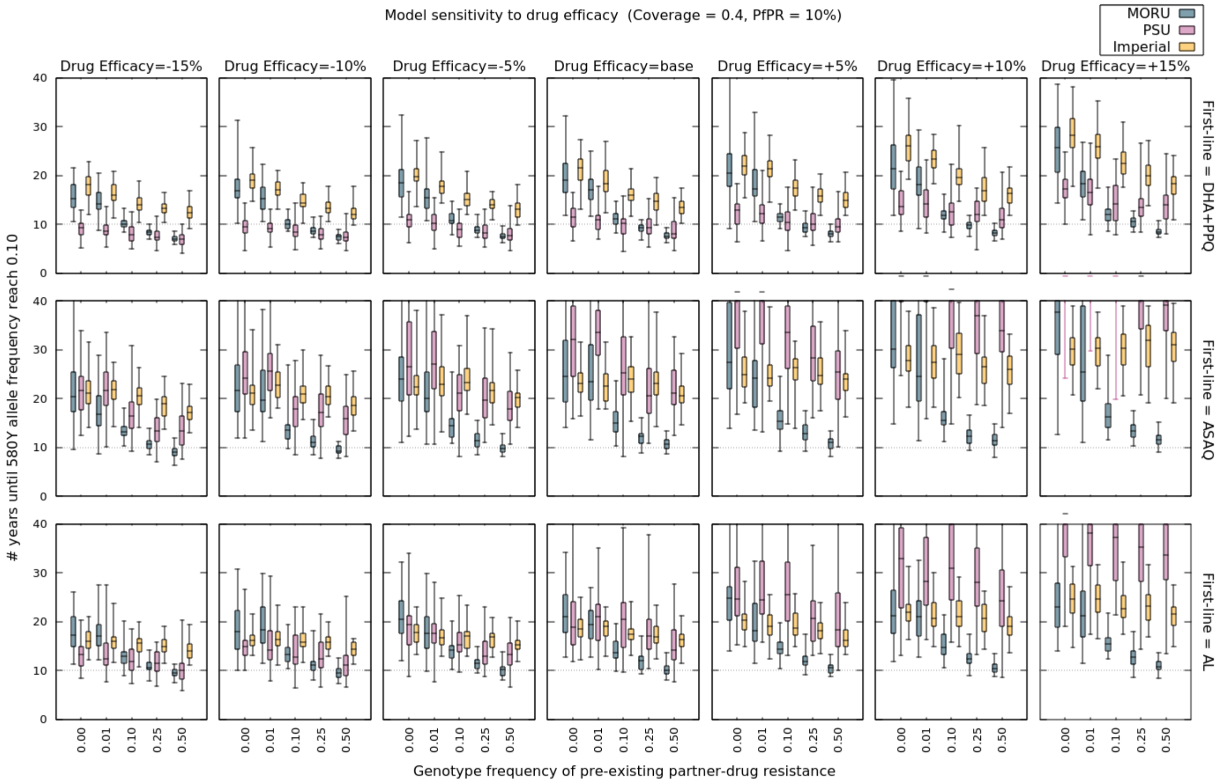

Supplementary Figure 9: Number or years until 580Y allele frequency to increase from 0.00 to 0.25 in regions depending on assumed drug efficacy. The box (median and interquartile range) and whisker (95% quantile range from 50 simulation realisations) plots presented are censored boxplots, with simulations only being run for 40 years. Values greater than 40 years contribute to the median, quartiles and 95% ranges if calculable.

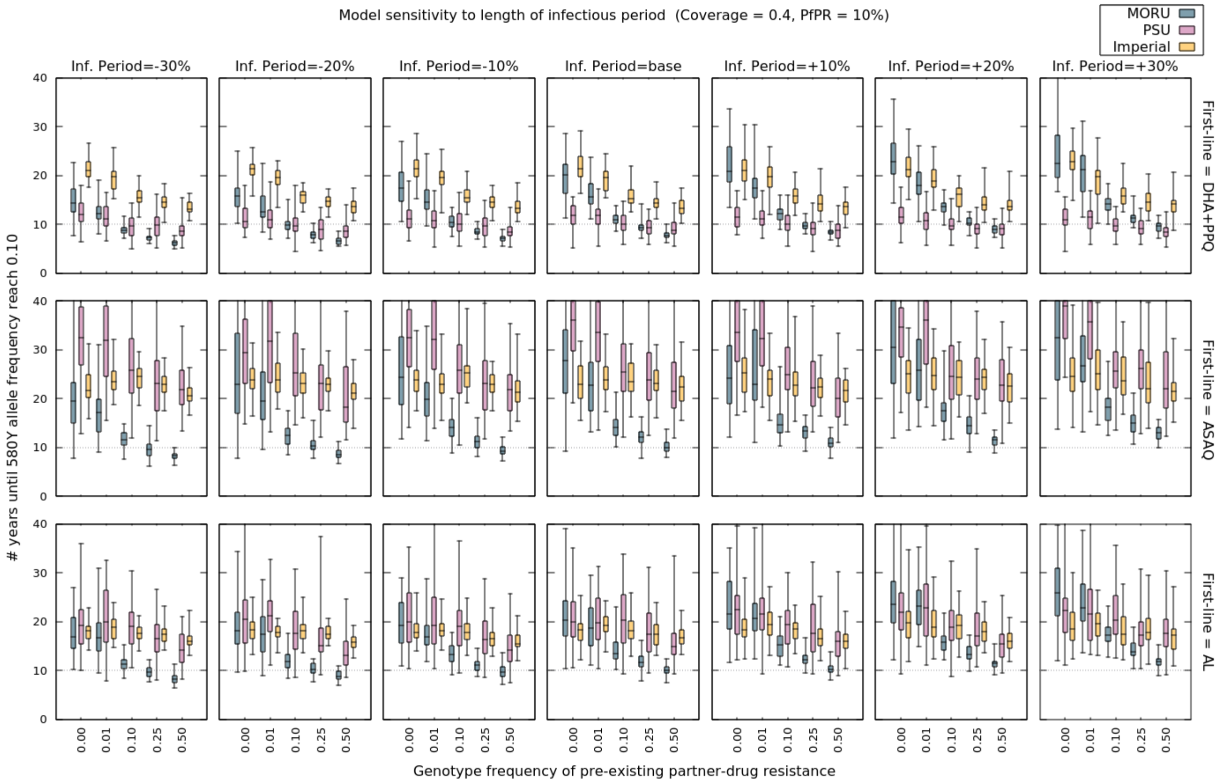

Supplementary Figure 10: Number or years until 580Y allele frequency to increase from 0.00 to 0.25 in regions depending on assumed infectious period. The box (median and interquartile range) and whisker (95% quantile range from 50 simulation realisations) plots presented are censored boxplots, with simulations only being run for 40 years. Values greater than 40 years contribute to the median, quartiles and 95% ranges if calculable.

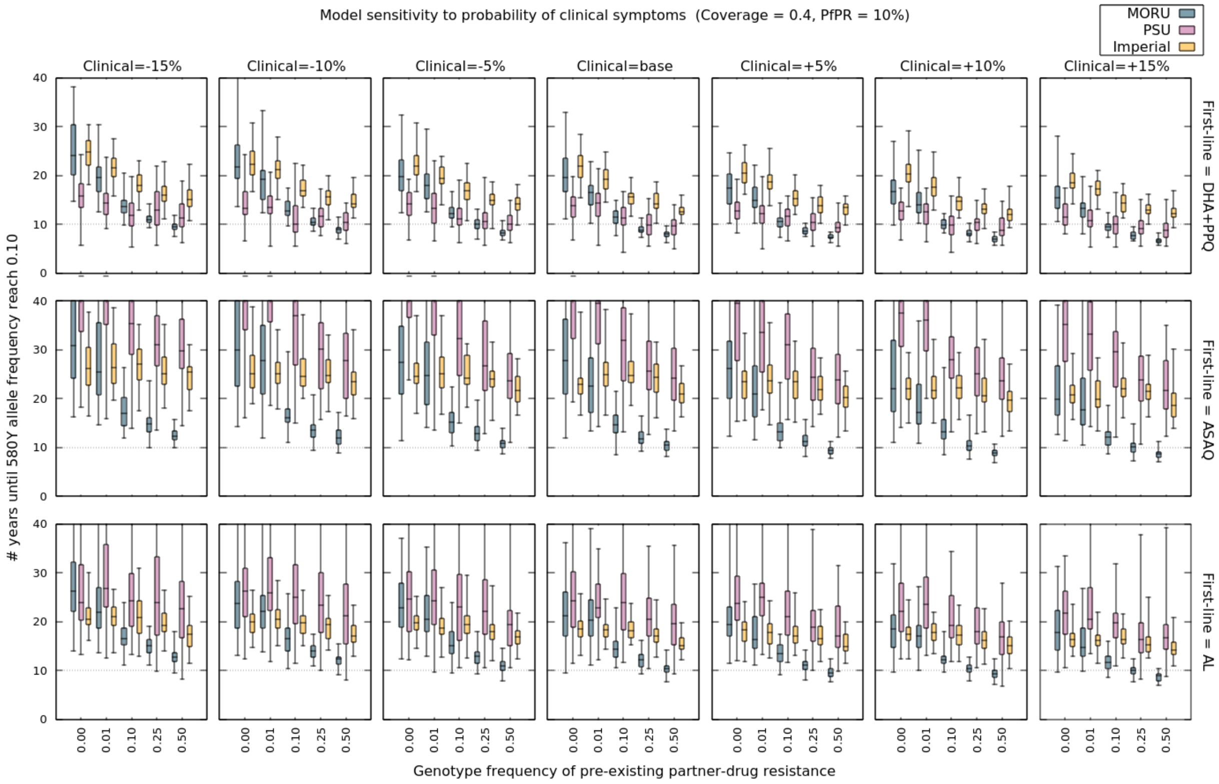

Supplementary Figure 11: Number or years until 580Y allele frequency to increase from 0.00 to 0.25 in regions depending on assumed probability of developing clinical symptoms. The box (median and interquartile range) and whisker (95% quantile range from 50 simulation realisations) plots presented are censored boxplots, with simulations only being run for 40 years. Values greater than 40 years contribute to the median, quartiles and 95% ranges if calculable.

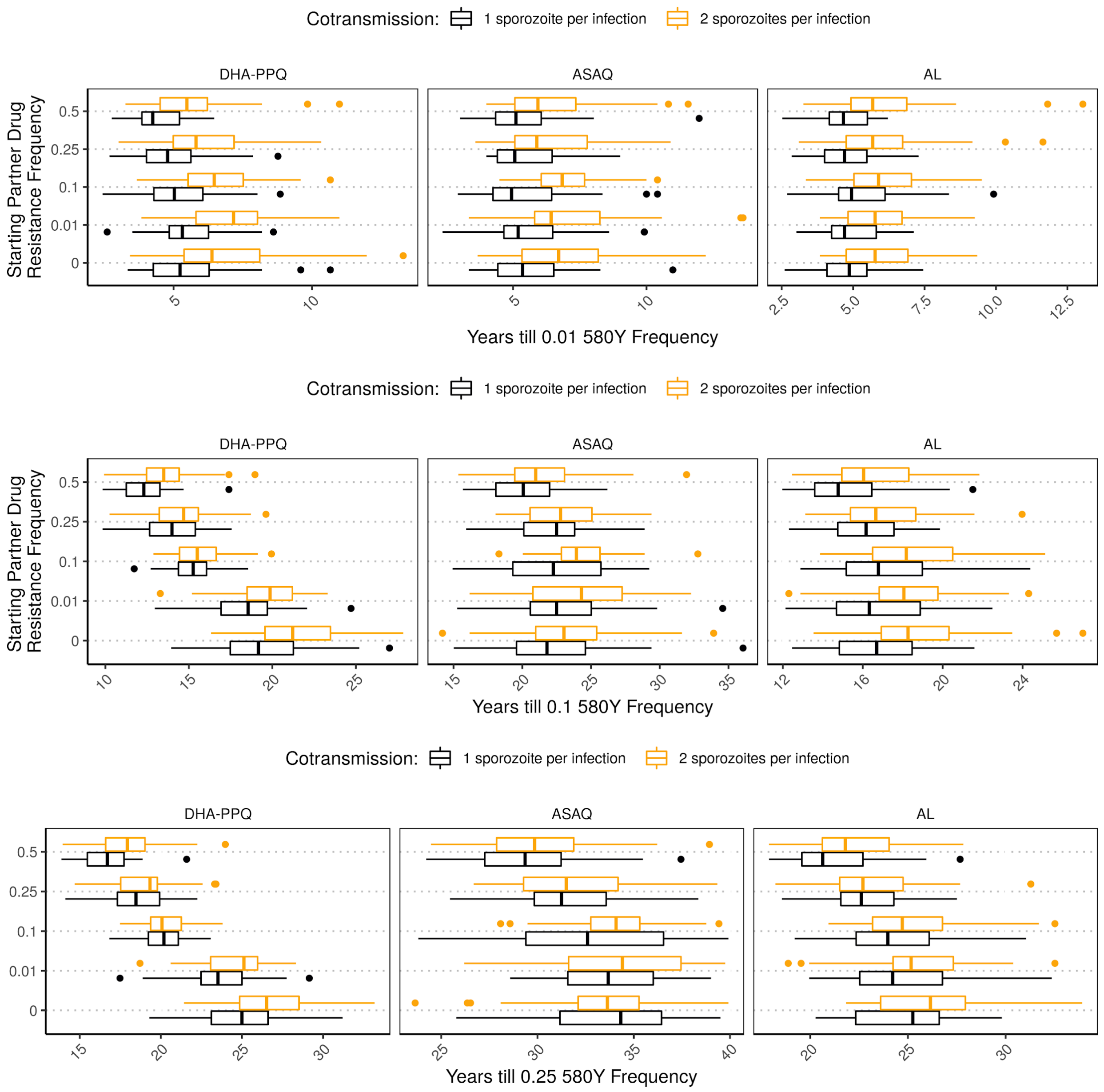

Supplementary Figure 12: Comparison of selection speed for AL and ASAQ under reduced cotransmission of sporozoites. The effect of reducing the frequency of cotransmission events (multiple sporozoites being transmitted from one feeding event) on the speed of selection in the Imperial model is shown for each drug strategy and drug resistance milestone. Reducing cotransmission increases selection due to the reduction in interclone competition manifested by a reduction in the probability that an emergent resistant parasite will be sampled when a polyclonally infected individual is bitten by a mosquito.

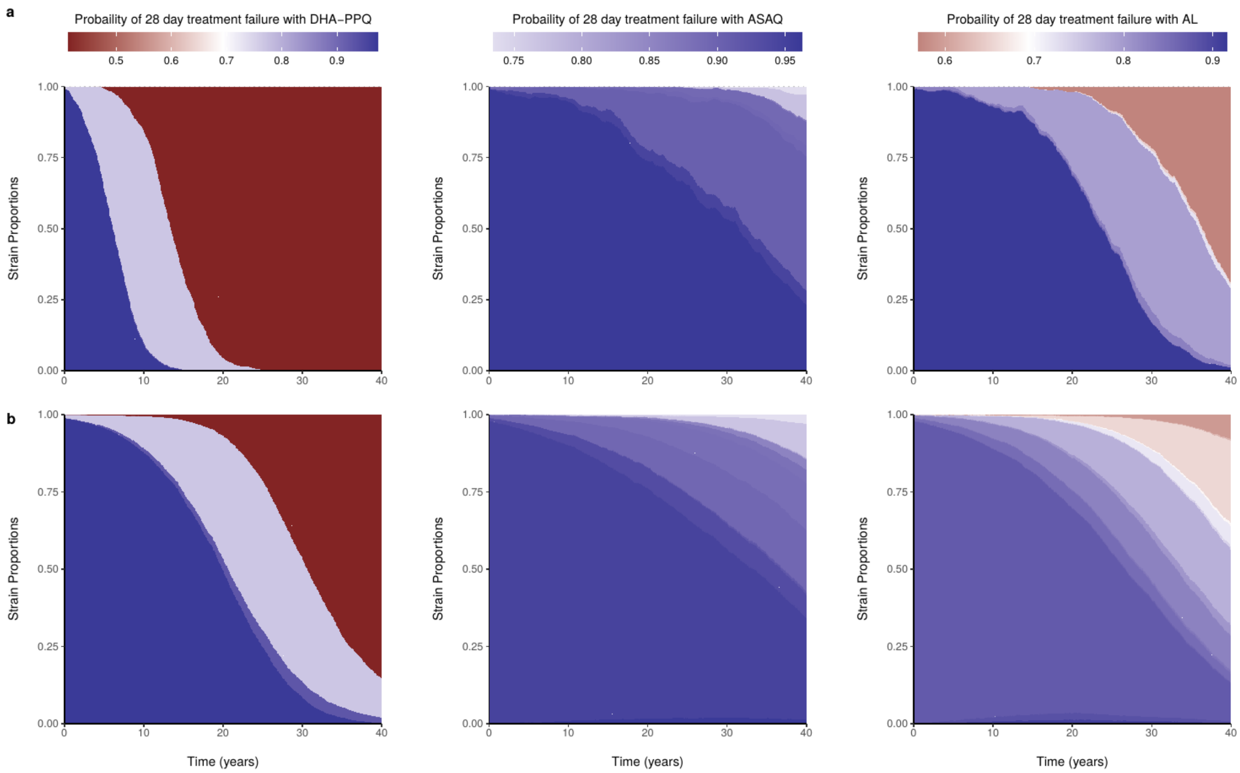

Supplementary Figure 13: Clonal interference differences dependent on mutation assumptions. The proportion of each strain is shown over time for one stochastic repetition of **a)** the PSU model and **b)** the Imperial model. The mean probability of treatment failure after 28-days for each strain is shown, indicating the increasing treatment failure over time as wildtype parasite strains (less resistant = blue) are replaced with resistant strains (more resistant = red). For treatment with AL and ASAQ, the increased complexity of the fitness landscape resulting from increased loci associated with partner drug resistance increases clonal interference between resistant strains. The assumed absence of mutations leading to less fit clones in the PSU model results in fewer clones being generated in the ASAQ and AL scenario reducing clonal interference in comparison to the Imperial model.

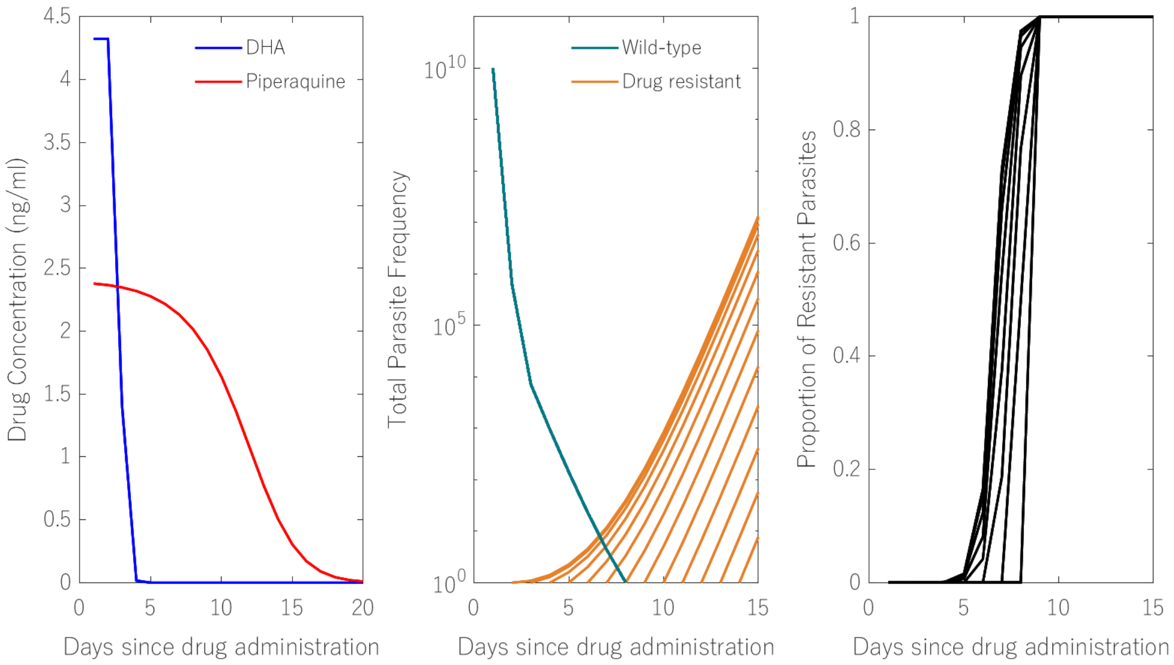

Supplementary Figure 14. Within host dynamics following a clinical infection treatment. Here, we depict the time dynamics of drug concentration (left), and parasite population frequencies (middle, right) after treatment with DHA-PPQ. The middle panel shows how drug resistant clones (in this case, resistant to both drugs) emerging at different points in time (each line is a different simulation) will eventually outcompete wild-type parasites.

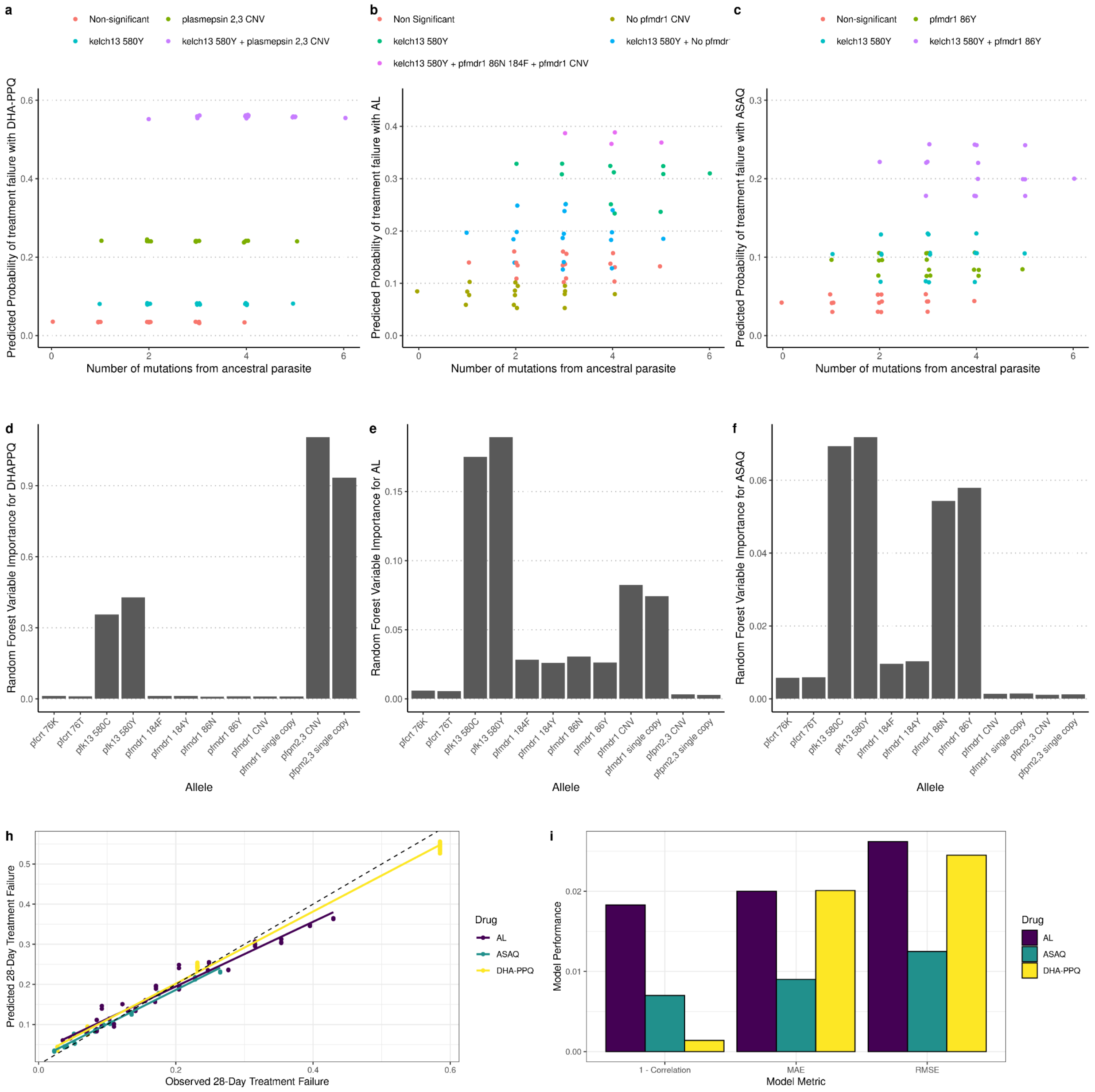

Supplementary Figure 15: Contribution of resistance alleles to treatment failure from random forest model. The predicted treatment failure from a random forest model for **a)** DHA-PPQ, **b)** AL and **c)** ASAQ is shown for each of the 64 genotypes modelled. The model was trained using the presence/absence of each allele to predict the treatment efficacy, with the presence of specific alleles shown by the colour of the points. In **d)**, **e)** and **f)** the importance of each allele towards the random forest is shown for each ACT. The variable importance is most defined for **d)** DHA-PPQ, for which only *pfkelch13* and *pfplasmepsion2,3* impact the resistance phenotype, whereas the contribution of each allele is more complex for **e)** AL and **f)** ASAQ. In **h)** the model predictions for each drug are compared against the observed 28-day treatment failure, with model performance metrics for each drug shown in **i)**.

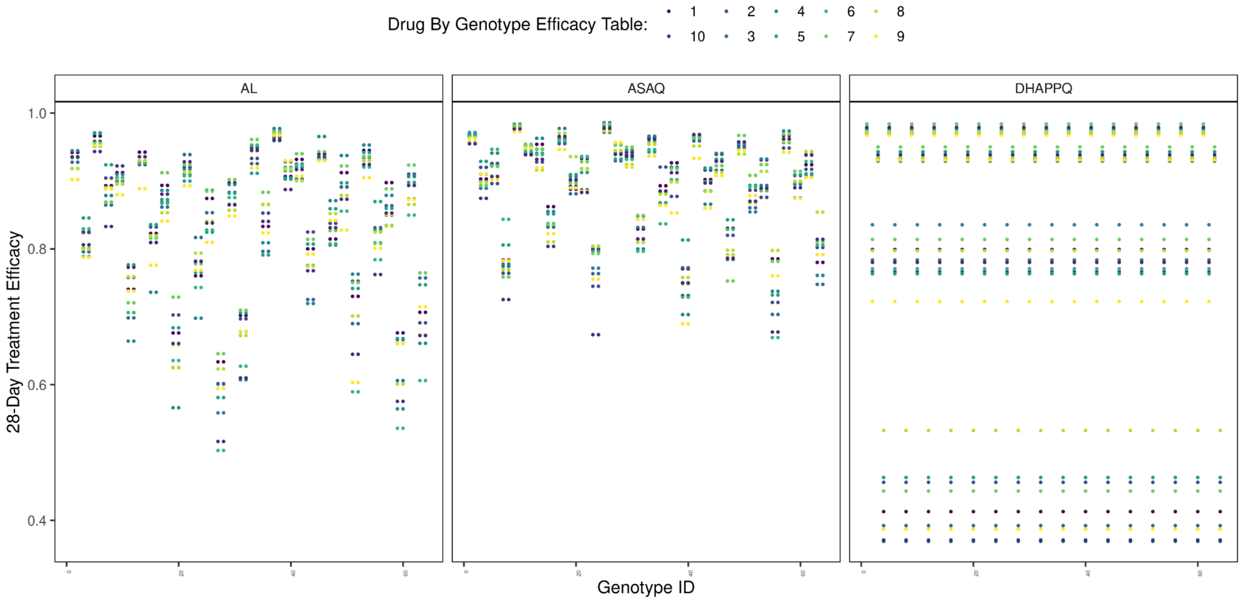

Supplementary Figure 16: Altered Drug by Efficacy Table Estimates. To explore the impact of uncertainty in the relationships between genotype and drug treatment efficacy, we explored ten additional drug by efficacy tables (see “5 Impact of altering relationship between genotypes and drug efficacy”). Each of the ten tables is shown with a different colour, with the 28-day treatment efficacy of each ACT shown for each of the 64 genotypes detailed in Supplementary Table 3. Each table was generated by drawing new estimates of the EC50 values for each drug component from a normal distribution with mean equal to the default EC50 value and standard deviation = 0.1, i.e. 10%.

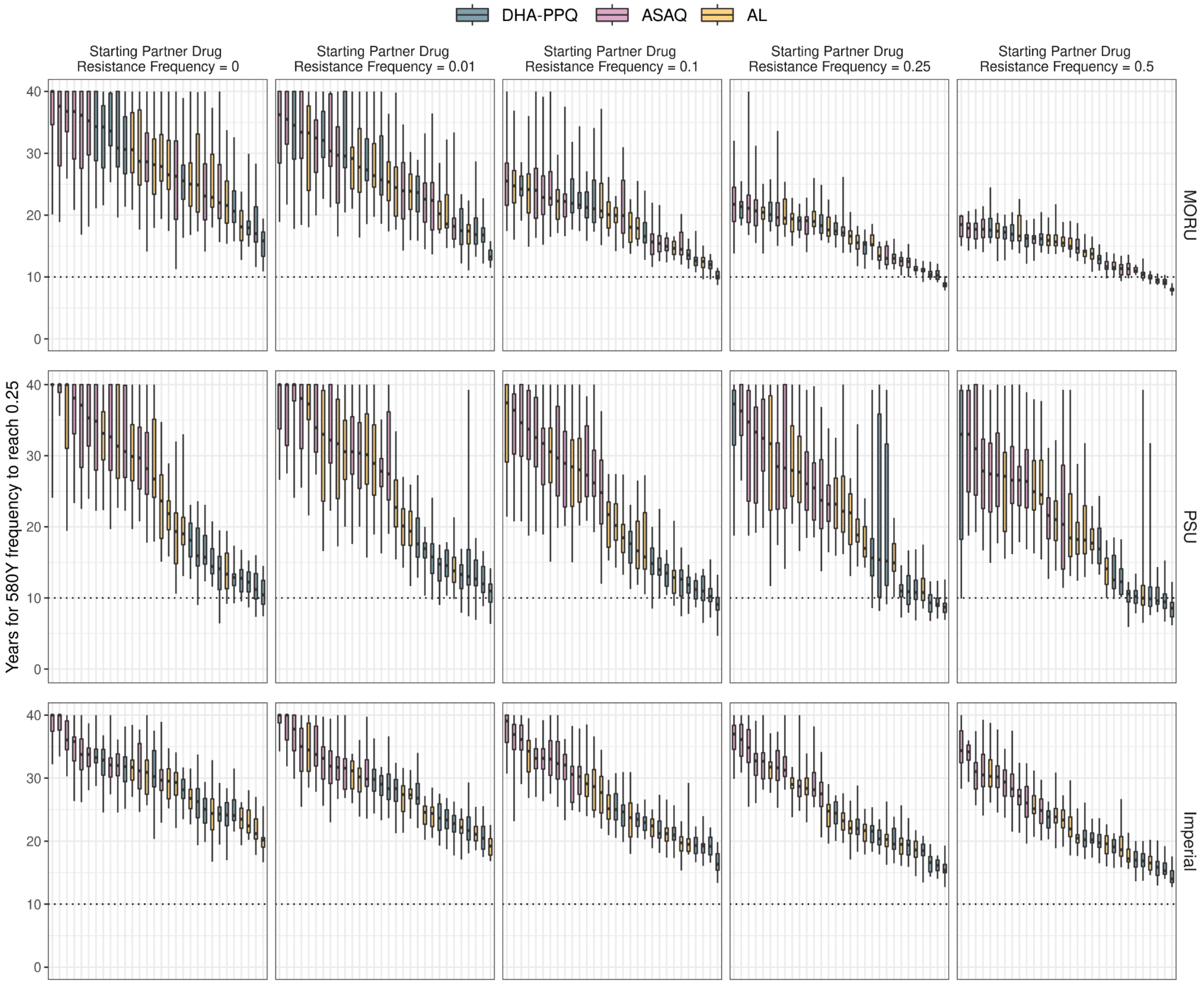

Supplementary Figure 17: Impact of Altered Drug by Efficacy Table Estimates. Number of years until 580Y allele frequency increases from 0.00 to 0.25 is shown for each of the 10 additional drug by efficacy tables explored (see “5.1 Uncertainty in estimates of drug efficacy by genotype” and Supplementary Figure 16). Each row shows the results for each model (MORU, PSU and Imperial), each column shows the results for a different starting partner drug resistance frequency (0, 0.01, 0.1, 0.25 and 0.5 from left to right), with the colour of the box indicating the ACT used. In each of the 15 subplots, 30 box and whiskers are shown representing the 3 ACTs used and the 10 different drug by genotype efficacy tables explored. In each of the subplots, the box and whiskers are ordered by the median number of years for 580Y frequency to increase from 0.00 to 0.25 (i.e. the ordering of each box and whisker can be different between the subplots). The ordering of the results in this way shows that for a number of the drug by genotype efficacy tables, we observe faster selection for AL compared to DHA-PPQ.

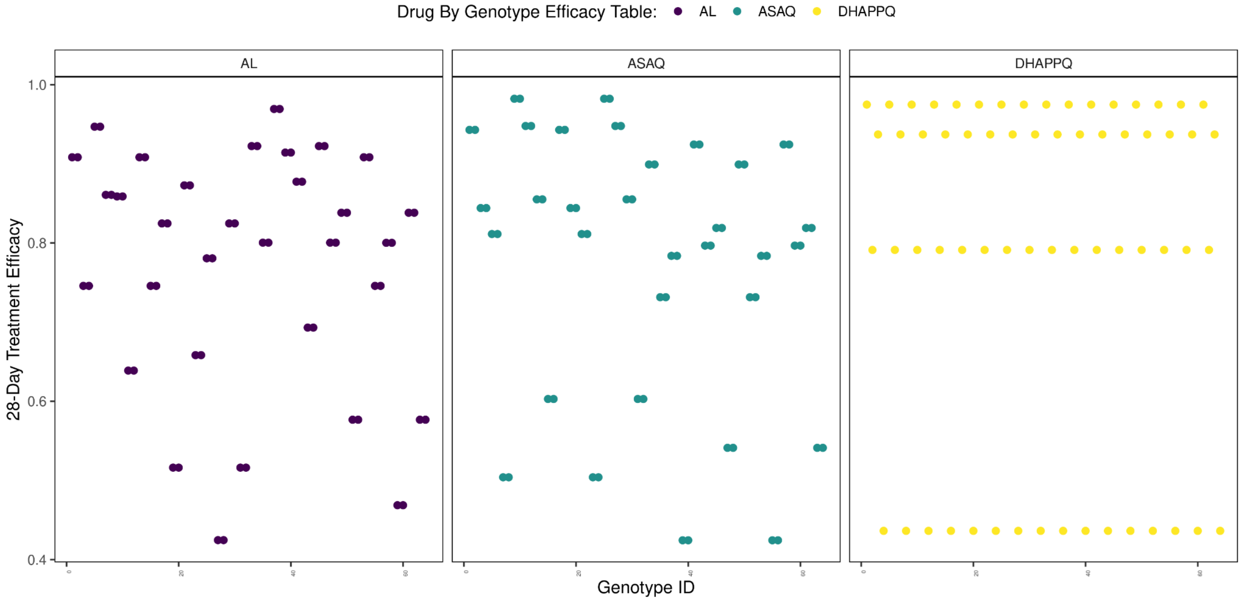

Supplementary Figure 18: Drug by genotype efficacy values under a scenario of comparable drug efficacies for each ACT. To explore the impact of the genetic landscape, we explored an additional drug by efficacy table (see "5.2 Comparable drug efficacy by genotype across ACT") in which the maximum and minimum efficacy for AL and ASAQ was set equal to DHA-PPQ. For the resultant table, the 28-day treatment efficacy of each ACT is shown for each of the 64 genotypes detailed in Supplementary Table 3.

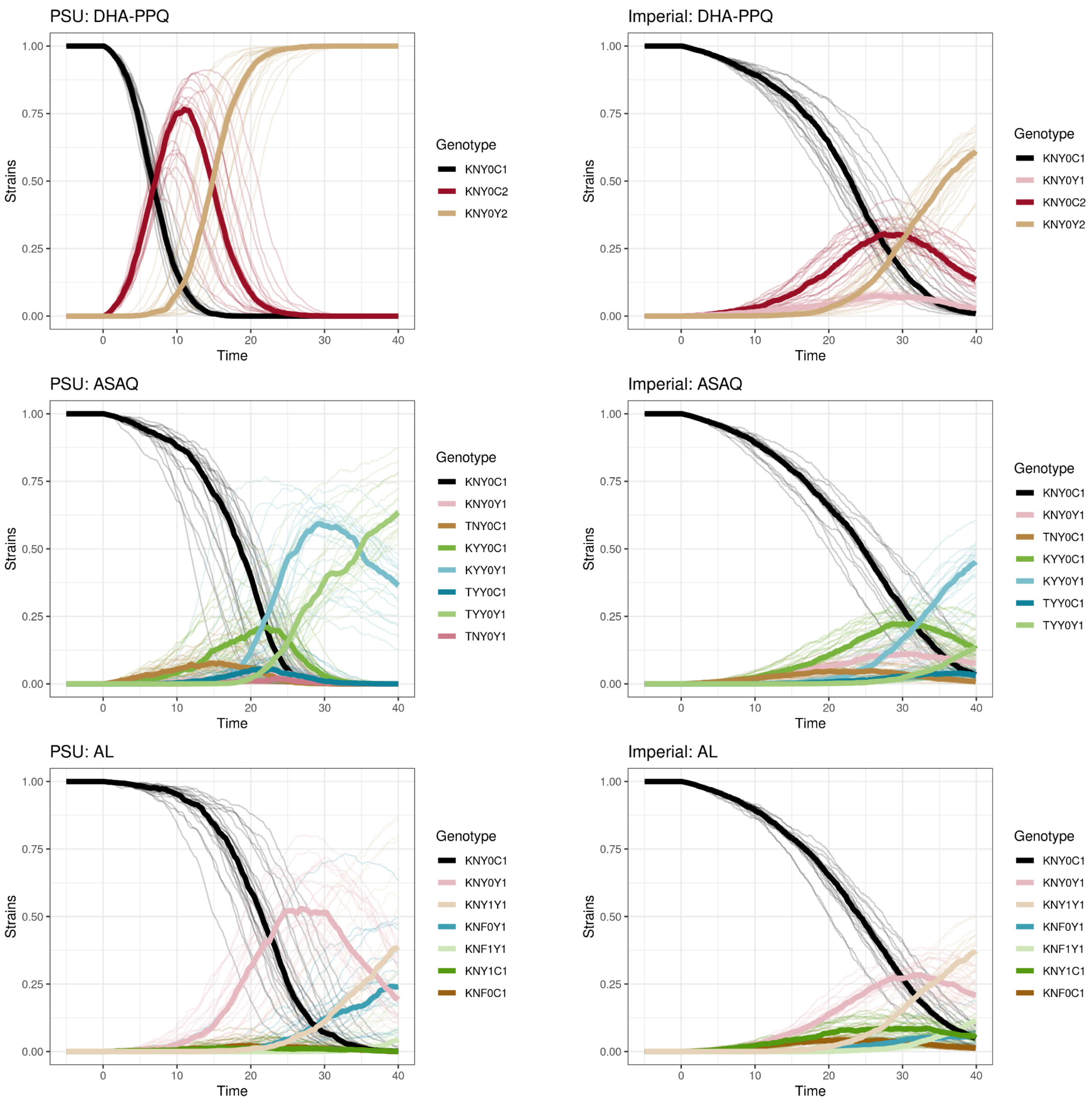

Supplementary Figure 19: Ordering of selection events when drug efficacy by genotype are comparable across ACTs. Using a drug by genotype efficacy table (Supplementary Figure 18) with the same minimum and maximum treatment efficacy across each ACT (see "5.2 Comparable drug efficacy by genotype across ACT"), the observed frequency of genotypes is shown for the PSU and Imperial model, in a scenario with no pre-existing partner drug resistance, 10% PfPR and 40% treatment coverage. In each plot, 20 simulation replicates are shown, with the median shown with a thick line. Only genotypes that reach at least a strain frequency of 0.05 in at least one simulation realisation are shown. These analyses demonstrate that DHA-PPQ continues to select most quickly for the maximally resistant genotype, reflecting the simpler mutational pathway for multidrug resistance to DHA-PPQ.

Supplementary Tables

Model	ACT	Years till 0.25 580Y frequency			
		PfPR = 1%	PfPR = 5%	PfPR = 10%	PfPR = 20%
MORU	DHA-PPQ	51.5 [36.7, 68.5]	25.6 [21.4, 32.2]	22.6 [19.4, 25.8]	23.5 [21.8, 25.3]
PSU	DHA-PPQ	19.6 [16.2, 22.3]	13.9 [11.7, 16.1]	13.4 [11.8, 15.5]	12.3 [10.6, 13.5]
Imperial	DHA-PPQ	36.3 [28.0, 41.9]	27.1 [24.8, 30.7]	26.0 [24.6, 28.2]	27.5 [26.4, 28.2]
MORU	ASAQ	64.5 [48.8, 80.1]	41.7 [30.5, 53.3]	28.9 [23.9, 37.7]	27.8 [25.6, 32.0]
PSU	ASAQ	46.7 [38.9, 54.0]	38.8 [31.0, 44.0]	38.4 [32.8, 43.2]	36.2 [32.1, 40.6]
Imperial	ASAQ	46.0 [30.1, 58.6]	38.3 [33.2, 42.0]	35.2 [32.5, 39.6]	35.1 [33.3, 36.9]
MORU	AL	39.8 [28.1, 49.7]	27.2 [22.4, 34.4]	24.5 [21.0, 28.2]	29.4 [26.3, 31.4]
PSU	AL	29.1 [20.3, 39.3]	24.5 [21.4, 30.8]	26.4 [23.0, 30.4]	26.8 [24.2, 30.3]
Imperial	AL	37.2 [26.4, 46.2]	28.0 [24.1, 31.7]	26.6 [24.9, 28.9]	26.1 [24.9, 27.3]

Supplementary Table 4. Time till 0.25 580Y frequency ($T_{Y0.25}$) under 40% treatment coverage and 0 starting partner drug resistance. Median and interquartile range (square brackets) is shown. Censored times above 40 years were inferred using a Weibull distribution to describe time-to-event values.

Supplementary Table 5. Times till 0.25 580Y frequency ($T_{Y0.25}$) under 40% treatment coverage and 1% PfPR under different starting partner drug (PD) resistance. Median years for scenarios with 0 starting partner drug resistance (PD_0) is given before showing the mean percentage difference in $T_{Y0.25}$ for each starting partner drug frequency explored. Range in square brackets reflects the interquartile range across all prevalence settings considered. Censored times above 40 years were inferred using a Weibull distribution to describe time-to-event values.

**Model**	**ACT**	**Years till 0.25 580Y frequency**	Percent reduction in establishment time (or artemisinin UTL) when comparing to **PD_0** scenario				**Years Lost per log_{10} PD increase**
		**$T_{Y0.25}$, PD_0**	**%$T_{Y0.25,}$ $PD_{0.01}$**	**%$T_{Y0.25,}$ $PD_{0.1}$**	**%$T_{Y0.25,}$ $PD_{0.25}$**	**%$T_{Y0.25,}$ $PD_{0.5}$**	
MORU	DHA-PPQ	51.5 [36.7, 68.5]	37.2% [25.6, 49.0]	67.1% [58.3, 76.1]	75.4% [67.0, 84.2]	78.9% [70.3, 87.6]	13.4
PSU	DHA-PPQ	19.6 [16.2, 22.3]	5.9% [-0.8, 13.1]	27.5% [20.8, 34.3]	32.6% [25.4, 39.6]	38.0% [31.5, 44.1]	3.6
Imperial	DHA-PPQ	36.3 [28.0, 41.9]	8.4% [1.3, 16.1]	30.2% [23.6, 37.0]	36.0% [28.9, 43.2]	39.9% [33.4, 45.9]	6.6
MORU	ASAQ	64.5 [48.8, 80.1]	7.9% [-3.6, 18.5]	55.8% [48.0, 64.2]	69.2% [62.4, 76.9]	78.8% [71.6, 86.0]	27.3
PSU	ASAQ	46.7 [38.9, 54.0]	4.7% [-3.0, 12.7]	23.5% [16.7, 31.0]	38.8% [32.3, 45.6]	34.9% [28.9, 41.8]	9.2
Imperial	ASAQ	46.0 [30.1, 58.6]	6.5% [-3.6, 16.5]	8.2% [-1.3, 17.3]	15.7% [6.4, 25.6]	18.6% [9.5, 27.2]	3.3
MORU	AL	39.8 [28.1, 49.7]	-3.5% [-14.7, 8.8]	44.4% [35.9, 52.8]	56.8% [48.7, 64.8]	65.2% [57.6, 72.9]	16.3
PSU	AL	29.1 [20.3, 39.3]	-4.2% [-15.2, 6.8]	16.4% [6.8, 26.7]	19.4% [9.6, 28.8]	31.6% [22.1, 40.9]	6
Imperial	AL	37.2 [26.4, 46.2]	13.0% [4.5, 21.2]	14.9% [6.7, 23.4]	18.0% [10.3, 26.2]	22.8% [14.3, 31.0]	1.9

**Supplementary Table 6. Times till 0.25 580Y frequency ($T_{Y0.25}$) under 40% treatment coverage and 10% PfPR under different starting partner drug (PD) resistance.** Median years for scenarios with 0 starting partner drug resistance (PD_0) is given before showing the mean percentage difference in $T_{Y0.25}$ for each starting partner drug frequency explored. Range in square brackets reflects the interquartile range across all prevalence settings considered. Censored times above 40 years were inferred using a Weibull distribution to describe time-to-event values.							
**Model**	**ACT**	**Years till 0.25 580Y frequency**	Percent reduction in establishment time (or artemisinin UTL) when comparing to **PD_0** scenario				**Years Lost per log_{10} PD increase**
		**$T_{Y0.25}$, PD_0**	**%$T_{Y0.25,}$ $PD_{0.01}$**	**%$T_{Y0.25,}$ $PD_{0.1}$**	**%$T_{Y0.25,}$ $PD_{0.25}$**	**%$T_{Y0.25,}$ $PD_{0.5}$**	
MORU	DHA-PPQ	22.6 [19.4, 25.8]	15.5% [10.1, 21.0]	37.0% [32.9, 41.4]	46.5% [42.7, 50.6]	51.0% [46.4, 55.4]	4.9
PSU	DHA-PPQ	13.4 [11.8, 15.5]	2.4% [-2.4, 7.1]	9.9% [5.1, 14.9]	15.7% [11.1, 20.3]	16.4% [12.1, 20.9]	1.2
Imperial	DHA-PPQ	26.0 [24.6, 28.2]	7.2% [4.3, 10.3]	22.3% [19.8, 25.1]	28.2% [25.3, 30.8]	30.2% [27.5, 32.7]	3.7
MORU	ASAQ	28.9 [23.9, 37.7]	12.2% [4.5, 19.6]	41.3% [35.8, 47.0]	51.0% [45.6, 56.6]	55.7% [50.1, 61.4]	8
PSU	ASAQ	38.4 [32.8, 43.2]	-0.4% [-4.9, 4.2]	12.7% [8.0, 17.7]	26.6% [21.8, 31.1]	32.9% [28.6, 37.4]	7.5
Imperial	ASAQ	35.2 [32.5, 39.6]	2.0% [-1.3, 5.0]	5.0% [2.1, 7.9]	8.1% [4.8, 11.1]	15.5% [12.5, 18.5]	2.5
MORU	AL	24.5 [21.0, 28.2]	8.5% [2.1, 13.9]	30.0% [25.0, 34.4]	39.5% [34.6, 44.0]	46.5% [41.9, 51.2]	5.7
PSU	AL	26.4 [23.0, 30.4]	2.5% [-3.6, 8.4]	14.9% [8.8, 21.0]	22.6% [16.6, 29.2]	29.6% [24.0, 35.0]	4.2
Imperial	AL	26.6 [24.9, 28.9]	2.8% [-0.2, 6.3]	6.9% [4.1, 9.6]	11.1% [8.3, 14.0]	16.5% [14.0, 19.2]	2

Supplementary Table 7. Times till 0.25 580Y frequency ($T_{Y0.25}$) under 40% treatment coverage and 20% PfPR under different starting partner drug (PD) resistance. Median years for scenarios with 0 starting partner drug resistance (PD_0) is given before showing the mean percentage difference in $T_{Y0.25}$ for each starting partner drug frequency explored. Range in square brackets reflects the interquartile range across all prevalence settings considered. Censored times above 40 years were inferred using a Weibull distribution to describe time-to-event values.

Model	ACT	Years till 0.25 580Y frequency	Percent reduction in establishment time (or artemisinin UTL) when comparing to PD_0 scenario				Years Lost per log_{10} PD increase
		$T_{Y0.25}$, PD_0	%$T_{Y0.25,}$ $PD_{0.01}$	%$T_{Y0.25,}$ $PD_{0.1}$	%$T_{Y0.25,}$ $PD_{0.25}$	%$T_{Y0.25,}$ $PD_{0.5}$	
MORU	DHA-PPQ	23.5 [21.8, 25.3]	8.2% [5.6, 10.9]	29.7% [27.4, 32.0]	37.9% [36.0, 40.0]	44.2% [42.1, 46.3]	5
PSU	DHA-PPQ	12.3 [10.6, 13.5]	1.1% [-3.2, 5.2]	6.0% [1.8, 9.9]	8.6% [4.6, 12.8]	10.7% [6.6, 14.7]	0.7
Imperial	DHA-PPQ	27.5 [26.4, 28.2]	8.4% [6.8, 10.1]	21.4% [19.8, 23.0]	27.5% [26.0, 28.9]	31.4% [29.9, 32.8]	3.7
MORU	ASAQ	27.8 [25.6, 32.0]	6.6% [2.2, 11.3]	28.5% [25.0, 31.8]	37.8% [34.4, 41.2]	45.2% [41.9, 48.6]	6.5
PSU	ASAQ	36.2 [32.1, 40.6]	1.9% [-2.0, 6.0]	13.2% [9.0, 17.6]	20.8% [17.1, 24.8]	27.6% [24.0, 31.3]	5.3
Imperial	ASAQ	35.1 [33.3, 36.9]	-0.8% [-2.8, 1.4]	1.7% [-0.3, 3.7]	5.3% [3.1, 7.3]	12.2% [10.3, 14.0]	2.3
MORU	AL	29.4 [26.3, 31.4]	5.5% [2.0, 9.0]	24.3% [21.5, 27.1]	34.7% [31.9, 37.3]	42.3% [39.6, 44.9]	6.2
PSU	AL	26.8 [24.2, 30.3]	5.7% [0.8, 10.2]	11.4% [6.8, 16.3]	20.9% [16.1, 25.1]	23.6% [19.4, 27.9]	2.9
Imperial	AL	26.1 [24.9, 27.3]	1.2% [-0.6, 3.0]	3.5% [1.4, 5.5]	8.0% [6.1, 9.7]	13.4% [11.7, 15.2]	1.7

Supplementary References

Aguas, Ricardo, Lisa J. White, Robert W. Snow, and M. Gabriela M. Gomes. 2008. "Prospects for Malaria Eradication in Sub-Saharan Africa." *PloS One* 3 (3): e1767.

Breiman, Leo. 2001. "Random Forests." *Machine Learning* 45 (1): 5–32.

Bretscher, M. T., P. Dahal, J. Griffin, K. Stepniewska, Ac Ghani, and P. J. Guerin. 2019. "A Pooled Analysis of the Duration of Chemoprophylaxis against Malaria after Treatment with Artesunate-Amodiaquine and Artemether-Lumefantrine." *medRxiv*. https://doi.org/10.1101/19002741.

Collins, W. E., and G. M. Jeffery. 1999. "A Retrospective Examination of Sporozoite- and Trophozoite-Induced Infections with Plasmodium Falciparum: Development of Parasitologic and Clinical Immunity during Primary Infection." *The American Journal of Tropical Medicine and Hygiene* 61 (1 Suppl): 4–19.

Cui, Liwang, Guiyun Yan, Jetsumon Sattabongkot, Yaming Cao, Bin Chen, Xiaoguang Chen, Qi Fan, et al. 2012. "Malaria in the Greater Mekong Subregion: Heterogeneity and Complexity." *Acta Tropica* 121 (3): 227–39.

Erhart, Annette, Duc Thang Ngo, Van Ky Phan, Thi Tinh Ta, Chantal Van Overmeir, Niko Speybroeck, Valerie Obsomer, et al. 2005. "Epidemiology of Forest Malaria in Central Vietnam: A Large Scale Cross-Sectional Survey." *Malaria Journal* 4 (December): 58.

Eyles, D. E., and M. D. Young. 1951. "The Duration of Untreated or Inadequately Treated Plasmodium Falciparum Infections in the Human Host." *Journal. National Malaria Society* 10 (4): 327–36.

Filipe, João A. N., Eleanor M. Riley, Christopher J. Drakeley, Colin J. Sutherland, and Azra C. Ghani. 2007. "Determination of the Processes Driving the Acquisition of Immunity to Malaria Using a Mathematical Transmission Model." *PLoS Computational Biology* 3 (12): e255.

Gao, Bo, Sompob Saralamba, Yoel Lubell, Lisa J. White, Arjen M. Dondorp, and Ricardo Aguas. 2020. "Determinants of MDA Impact and Designing MDAs towards Malaria Elimination." *eLife* 9 (April). https://doi.org/10.7554/eLife.51773.

Griffin, Jamie T., Samir Bhatt, Marianne E. Sinka, Peter W. Gething, Michael Lynch, Edith Patouillard, Erin Shutes, et al. 2016. "Potential for Reduction of Burden and Local Elimination of Malaria by Reducing Plasmodium Falciparum Malaria Transmission: A Mathematical Modelling Study." *The Lancet Infectious Diseases* 3099 (15): 1–8.

Griffin, Jamie T., Neil M. Ferguson, and Azra C. Ghani. 2014. "Estimates of the Changing Age-Burden of Plasmodium Falciparum Malaria Disease in Sub-Saharan Africa." *Nature Communications* 5: 3136.

Griffin, Jamie T., T. Deirdre Hollingsworth, Lucy C. Okell, Thomas S. Churcher, Michael White, Wes Hinsley, Teun Bousema, et al. 2010. "Reducing Plasmodium Falciparum Malaria Transmission in Africa: A Model-Based Evaluation of Intervention Strategies." *PLoS Medicine* 7 (8). https://doi.org/10.1371/journal.pmed.1000324.

Griffin, Jamie T., T. Déirdre Hollingsworth, Hugh Reyburn, Chris J. Drakeley, Eleanor M. Riley, and Azra C. Ghani. 2015. "Gradual Acquisition of Immunity to Severe Malaria with Increasing Exposure." *Proceedings of the Royal Society B: Biological Sciences* 282: 20142657.

Gryseels, Charlotte, Lies Durnez, René Gerrets, Sambunny Uk, Sokha Suon, Srun Set, Pisen Phoeuk, et al. 2015. "Re-Imagining Malaria: Heterogeneity of Human and Mosquito Behaviour in Relation to Residual Malaria Transmission in Cambodia." *Malaria Journal* 14

(April): 165.
Maire, Nicolas, Thomas Smith, Amanda Ross, Seth Owusu-Agyei, Klaus Dietz, and Louis Molineaux. 2006. “A Model for Natural Immunity to Asexual Blood Stages of Plasmodium Falciparum Malaria in Endemic Areas.” *The American Journal of Tropical Medicine and Hygiene* 75 (2 Suppl): 19–31.
Marsh, K., and R. Snow. 1999. “Malaria Transmission and Morbidity.” *Parassitologia* 41: 241–46.
Molineaux, L., and G. Gramiccia. 1980. “The Garki Project. Research on the Epidemiology and Control of Malaria in the Sudan Savanna of West Africa.” https://doi.org/10.1016/0035-9203(81)90085-7.
Nguyen, Tran Dang, Piero Olliaro, Arjen M. Dondorp, J. Kevin Baird, Ha Minh Lam, Jeremy Farrar, Guy E. Thwaites, Nicholas J. White, and Maciej F. Boni. 2015. “Optimum Population-Level Use of Artemisinin Combination Therapies: A Modelling Study.” *The Lancet Global Health* 3 (12): e758–66.
Nguyen, Tran Dang, Thu Nguyen-Anh Tran, Daniel M. Parker, Nicholas J. White, and Maciej F. Boni. 2021. “Antimalarial Mass Drug Administration in Large Populations and the Evolution of Drug Resistance.” *bioRxiv*. https://doi.org/10.1101/2021.03.08.434496.
Okell, Lucy C., Matthew Cairns, Jamie T. Griffin, Neil M. Ferguson, Joel Tarning, George Jagoe, Pierre Hugo, et al. 2014. “Contrasting Benefits of Different Artemisinin Combination Therapies as First-Line Malaria Treatments Using Model-Based Cost-Effectiveness Analysis.” *Nature Communications* 5: 5606.
Owusu-Agyei, S., T. Smith, H-P Beck, L. Amenga-Etego, and I. Felger. 2002. “Molecular Epidemiology of Plasmodium Falciparum Infections among Asymptomatic Inhabitants of a Holoendemic Malarious Area in Northern Ghana.” *Tropical Medicine & International Health: TM & IH* 7 (5): 421–28.
Reyburn, Hugh, Redempta Mbatia, Chris Drakeley, Jane Bruce, Ilona Carneiro, Raimos Olomi, Jonathan Cox, et al. 2005. “Association of Transmission Intensity and Age with Clinical Manifestations and Case Fatality of Severe Plasmodium Falciparum Malaria.” *JAMA: The Journal of the American Medical Association* 293 (12): 1461–70.
Ross, Amanda, Gerry Killeen, and Thomas Smith. 2006. “Relationships between Host Infectivity to Mosquitoes and Asexual Parasite Density in Plasmodium Falciparum.” *The American Journal of Tropical Medicine and Hygiene* 75 (2 Suppl): 32–37.
Rowe, A., S. Y. Rowe, R. W. Snow, E. L. Korenromp, J. R. Armstrong-Schellenberg, C. Stein, B. L. Nahlen, J. Bryce, R. E. Black, and R. W. Steketee. 2006. “The Burden of Malaria Mortality among African Children in the Year 2000.” *International Journal of Epidemiology* 35 (3): 691–704.
Saralamba, S., W. Pan-Ngum, R. J. Maude, S. J. Lee, J. Tarning, N. Lindegardh, K. Chotivanich, et al. 2011. “Intrahost Modeling of Artemisinin Resistance in Plasmodium Falciparum.” *Proceedings of the National Academy of Sciences of the United States of America* 108 (1): 397–402.
Slater, Hannah C., Jamie T. Griffin, Azra C. Ghani, and Lucy C. Okell. 2016. “Assessing the Potential Impact of Artemisinin and Partner Drug Resistance in Sub - Saharan Africa.” *Malaria Journal*, 1–11.
Smith, D. L., J. Dushoff, R. W. Snow, and S. I. Hay. 2005. “The Entomological Inoculation Rate and Plasmodium Falciparum Infection in African Children.” *Nature* 438 (7067): 492–95.
Watson, O. J., Robert Verity, and Rich FitzJohn. 2021. *OJWatson/magenta: Release Magenta 1.3.0*. https://doi.org/10.5281/zenodo.4620137.
Watson, Oliver J., Lucy C. Okell, Joel Hellewell, Hannah C. Slater, H. Juliette T. Unwin, Irene

Omedo, Philip Bejon, et al. 2021. “Evaluating the Performance of Malaria Genetics for Inferring Changes in Transmission Intensity Using Transmission Modeling.” *Molecular Biology and Evolution* 38 (1): 274–89.
